# Human tripartite cortical network model for temporal assessment of alpha-synuclein aggregation and propagation in Parkinson’s Disease

**DOI:** 10.1038/s41531-024-00750-x

**Published:** 2024-07-28

**Authors:** Fikret Emre Kapucu, Iisa Tujula, Oskari Kulta, Lassi Sukki, Tomi Ryynänen, Hjalte Gram, Valtteri Vuolanto, Andrey Vinogradov, Joose Kreutzer, Poul Henning Jensen, Pasi Kallio, Susanna Narkilahti

**Affiliations:** 1 Neuro Group, Faculty of Medicine and Health Technology, Tampere University, Tampere, Finland; 2 Micro- and Nanosystems Research Group, Faculty of Medicine and Health Technology, Tampere University, Tampere, Finland; 3 Danish Research Institute of Translational Neuroscience – DANDRITE and Department of Biomedicine, Aarhus University, Aarhus, Denmark

**Keywords:** Parkinson's disease, Neurological models

## Abstract

Previous studies have shown that aggregated alpha-synuclein (α-s) protein, a key pathological marker of Parkinson’s disease (PD), can propagate between cells, thus participating in disease progression. This prion-like propagation has been widely studied using in vivo and in vitro models, including rodent and human cell cultures. In this study, our focus was on temporal assessment of functional changes during α-s aggregation and propagation in human induced pluripotent stem cell (hiPSC)-derived neuronal cultures and in engineered networks. Here, we report an engineered circular tripartite human neuronal network model in a microfluidic chip integrated with microelectrode arrays (MEAs) as a platform to study functional markers during α-s aggregation and propagation. We observed progressive aggregation of α-s in conventional neuronal cultures and in the exposed (proximal) compartments of circular tripartite networks following exposure to preformed α-s fibrils (PFF). Furthermore, aggregated forms propagated to distal compartments of the circular tripartite networks through axonal transport. We observed impacts of α-s aggregation on both the structure and function of neuronal cells, such as in presynaptic proteins, mitochondrial motility, calcium oscillations and neuronal activity. The model enabled an assessment of the early, middle, and late phases of α-s aggregation and its propagation during a 13-day follow-up period. While our temporal analysis suggested a complex interplay of structural and functional changes during the in vitro propagation of α-s aggregates, further investigation is required to elucidate the underlying mechanisms. Taken together, this study demonstrates the technical potential of our introduced model for conducting in-depth analyses for revealing such mechanisms.

## Introduction

One of the most well-known pathological features of alpha-synucleinopathies, including PD, is Lewy bodies/neurites, consisting of a large amount of accumulated, misfolded assemblies of a specific protein, α–s. This pathology is detected in postmortem brains of PD patients regardless of familial or sporadic cause. The initiation mechanisms of the process are not clear. It is known that α-s is phosphorylated and accumulates in insoluble forms^1,2^, and aggregated α-s recruits soluble proteins and promotes self-amplification^3^, followed by propagation to other neurons, initiating the same pathological process. α–s is mainly located in synapses and participates in synaptic transmission by binding to synaptic vesicles^4^. In pathological situations, it gains a toxic function and/or loses its normal function, leading to disturbances in synaptic and mitochondrial functionality, altering Ca^2+^ homeostasis and inducing oxidative stress and eventually neuronal death^5–8^.

In recent years, the aggregation and propagation of α–s has been commonly addressed within in vivo and in vitro PD models by utilizing PFFs as seeds to initiate α–s pathology^9,10^. Although in vivo animal models provide a natural ground to study disease progression, in vitro models allow flexibility in experimental design and the possibility to work in a more relevant human context by using hiPSC-derived neurons. In vitro studies delve into the examination of neurons from various brain regions, considering their respective locations and functional classifications in relation to the progression of α–s pathology. Some of the previous works focused on cortical networks to study PFF-seeded pathological α–s propagation^11–14^ due to the relevance of the cortex in various stages of sporadic PD^15,16^. Additionally, an earlier study investigated in more detail the role of pathological α–s aggregation on excitatory glutamatergic human cortical neurons^14^.

Recent uses of microfluidics have enabled more thorough development of disease propagation patterns with the advantage of controlling and guiding the model by controlling the axonal transfer of PFFs, isolating synaptically connected neurons and observing PFF-triggered aggregation^12–14,17,18^. These state-of-the-art models are progressive alternatives to conventional in vitro cultures that are not sufficient to represent the propagation path^19^. However, the currently used PD models with microfluidics lack integrated functional measurement systems, causing limitations for noninvasive temporal experiments^12–14,17,18,20^. Thus, neuronal activity has not been thoroughly assessed or associated with the process of α–s aggregation and propagation spatiotemporally. Previously, calcium oscillations were used to assess neuronal activity during α–s aggregation mainly in conventional culturing platforms^13,14,21,22^. MEA provides direct neuron-electrode contact as a noninvasive method for repeated electrophysiological recordings and spatiotemporal analysis and has been used to assess neuronal activity during α–s aggregation in conventional cultures^11,23–25^. In summary, previous studies focusing on the functional aspects of PFF-induced α–s aggregation were performed on neuronal cultures from different species with different timelines and in different phases of aggregation with varying results^11,13,14,21–25^. For this reason, new insights combining microfluidic technology with MEAs^19,26^ are needed to provide more comprehensive information on the pathophysiological progress of axonal propagation of aggregated α–s and enable a more robust timeline assessment of its effects on the progression of PD.

Here, we performed studies both in conventional and engineered human cortical networks to evaluate the effects of PFF-induced α–s aggregation and propagation on structural and functional levels with 13 days of follow-up. The results showed the loss of presynaptic proteins as well as alterations in the calcium oscillation features and mitochondrial motility during different phases of α–s aggregation. Moreover, we propose an α-s aggregation/propagation model in a circular tripartite network with embedded MEAs and report the spatiotemporal effects from the early to late phases of PFF-seeded α-s aggregation on the functioning of hiPSC-derived cortical neurons. Briefly, in vitro neuronal activity changes from proximal to distal regions were tracked temporally, and a consistency in reduced activity was reported. In addition to the decrease in neuronal activity, glutamatergic receptor-modulated neuronal activity exhibited moderate distinct features in healthy neuronal networks and in neuronal networks containing α-s aggregates in the same circular tripartite network model, as revealed by multiparametric analysis.

## Results

### hiPSC-derived cortical networks have synaptic connections with developed neuronal activity and express α-s

**Fig. 1:**
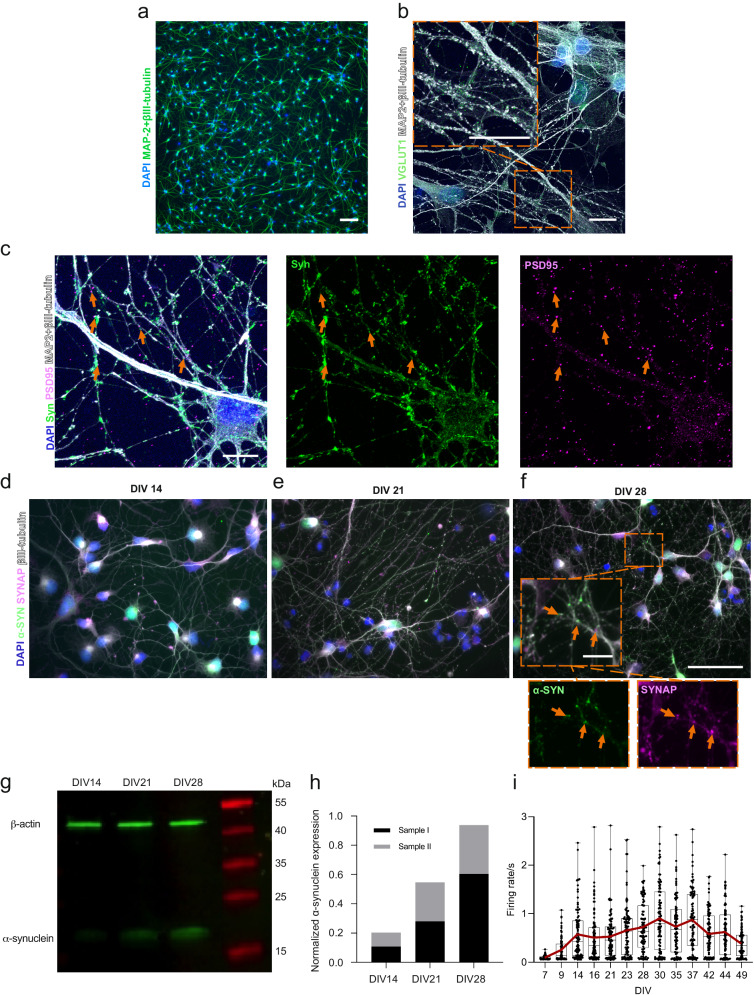
Maturation of hiPSC-derived cortical networks. **a** Display of neuronal cells possessing axonal and dendritic extensions at DIV 14. Scale bar represents 100 µm. **b** A representative image showcases the expression of the glutamatergic marker vGlut1 (depicted as green puncta) in hiPSC-derived cortical neurons at DIV 35. It is primarily localized within neurites, indicated by MAP2 and βIII-tubulin. Scale bars represent 10 µm. **c** Representative image illustrating the colligation of the pre-synaptic marker synaptophysin (Syn, green) and the post-synaptic marker PSD95 (magenta), with orange arrowheads pointing out their spatial convergence. Scale bar denotes 10 µm. **d** Visualization of the endogenous expression of α-s (green) in hiPSC-derived neurons at DIV 14, **e** DIV 21 and **f** DIV 28. The orange arrowheads highlight the colocalization of synaptophysin with presynaptic markers, SYNAP (synaptophysin and synapsin1) at DIV 28. Scale bars are 50 µm and 10 µm (inset). **g** Western blot of total endogenous α-s and β-actin and **h** quantification of total endogenous α-s relative to total β-actin based on two independent samples from DIV 14 to DIV 28. **i** Representation of neuronal activity by means of mean firing rate, recorded from hiPSC-derived neuronal cultures in conventional MEA wells (*n* = 93) between DIV 7 and DIV 49. Data points indicate the average values of each well. The range and median values are depicted by whiskers and horizontal lines, respectively, with a red line indicating the trend in average values.

We have used the same hiPSC line for cortical neuronal differentiation in our previous work^27^ and have used the protocol in several studies with their characterization included^28^. The maturity of the neurons was followed from Day 32 onward of neuronal differentiation (accounting for DIV 0), when neural progenitor cells were plated for the final experiments (Supplementary Fig. 1). In the following days, neurons formed extended networks (Fig. 1a). Endogenous expression of α-s was investigated between DIV 14 and 28 to ensure its expression in neurons and presynaptic localization prior to PFF seeding experiments. The expression of α-s in neuronal somas on DIV 14 (Fig. 1d) gradually expanded toward the synapses on DIV 21 (Fig. 1e) and DIV 28 (Fig. 1f). The presynaptic proteins synaptophysin and synapsin 1 together (SYNAP) colocalized with α-s on DIV 21 and 28 when more puncta-like presynaptic proteins were evident (Fig. 1e, f). A gradual increase in α-s expression from DIV 14 to DIV 28 was also observed by Western blot (Fig. 1g) and quantified (Fig. 1h). The glutamatergic marker vesicular glutamate transporter 1 (vGlut1) was widely expressed in neurons on DIV 35 (Fig. 1b), and cultures also contained GAD67-positive GABAergic neurons (Supplementary Fig. 2). Structural synaptic connections were revealed with the colligation of pre- and postsynaptic markers (synaptophysin and PSD95, respectively, Fig. 1c). Neuronal activity was recorded by means of extracellular action potentials that could be silenced with the application of the Na^+^ channel blocker tetrodotoxin (TTX) (Supplementary Fig. 3a). The development of neuronal activity was followed up until the PFF treatment day, DIV 49, on conventional MEAs (Supplementary Fig. 1a). Median activity reached its peak during DIV 37; however, a progressive increase in neuronal activity in some of the wells was observed until DIV 42 (Fig. 1i).

### Engineered circular tripartite and axonal elongation cortical models isolate neuronal soma and axons to separated compartments

**Fig. 2:**
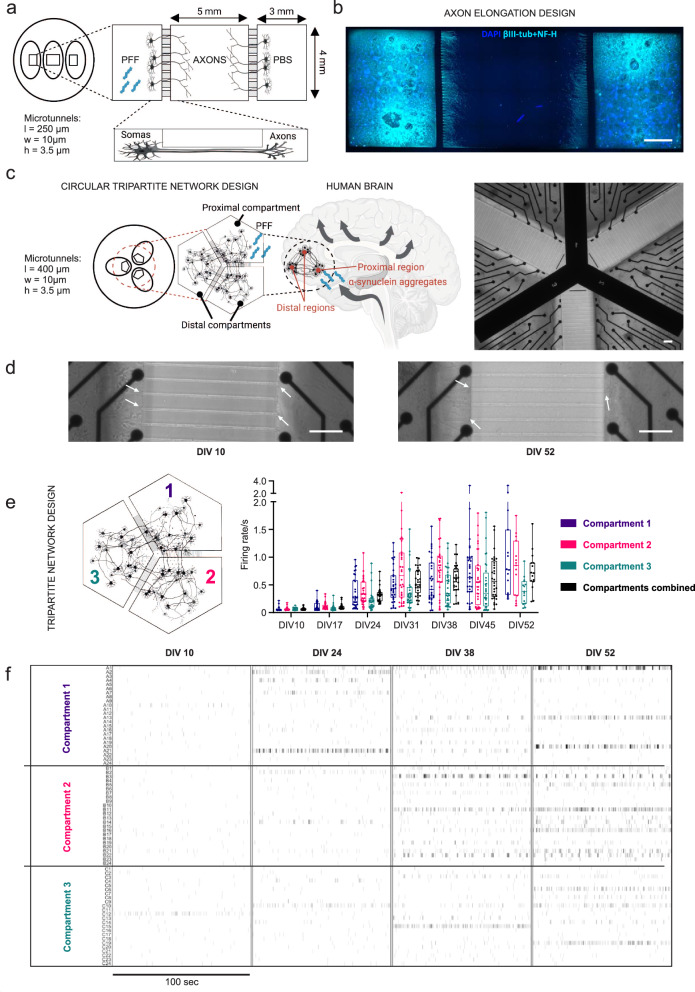
Engineered circular tripartite and axon elongation cortical models. **a** Schematic of the axon elongation design to study propagation of α-s aggregates, consisting of two side compartments (3 × 4 mm) and a central compartment (5 × 4 mm) connected via microtunnels (250 × 10 × 3.5 mm). One side compartment is allocated for PFF treatment and the other for PBS control. **b** Immunostaining shows axon elongation using NF-H and βIII-tubulin together (light blue) on DIV 14. Axons from side compartments are distinct in the central area. Scale bar represents 1 mm. **c** Schematic of the circular tripartite network design, mimicking neuronal connectivity in the cortex, to study neuronal functionality during the propagation of α-s aggregates from proximal to distal regions. The design entails three interconnected compartments forming a circular network, connected via microtunnels (400 × 10 × 3.5 mm). Phase contrast image displays compartment configuration and electrode layout. Scale bar represents 100 µm. **d** Axonal extensions from individual axons (white arrows) developing into thick bundles towards microtunnels from DIV 10 to DIV 52 in the circular tripartite network. Scale bar represents 100 µm. **e** Mean firing rates of hiPSC-derived neurons in individual compartments of the tripartite design from DIV 10 to DIV 52 and **f** their representative raster plots showing neuronal activity progression in these compartments. Data points in (**e**) represents the average values of each compartment. Whiskers and horizontal lines indicate the range and the median values, respectively. All illustrations were generated using Biorender.com.

Cortical networks were cultured not only in conventional culture wells but also in compartmentalized designs to develop more accurate in vitro models of α–s propagation. Isolation of somas and axons to separate compartments for different purposes was achieved with the two different cortical network designs developed in-house (axonal elongation^29,30^ and circular tripartite design^31^) with the same microtunnel width (w) and height (h) but different lengths (l) (Fig. 2a, c). An axonal elongation design (Fig. 2a) was used for testing different concentrations of PFF and optimizing the propagation of aggregated forms of α–s via axonal transport. Side compartments of axonal elongation design were utilized for somatic isolation, and the middle compartment was utilized for investigating axonal extensions (Fig. 2a). This setup allowed us to investigate the effects of PFF and vehicle (PBS) treatment under the same conditions. A circular tripartite design (Fig. 2c) was used to establish a circular tripartite network and model PFF-induced aggregation and propagation in the paradigm of cortex with impacts on neuronal activity.

Axonal extensions from both side compartments were visible in the middle compartment of the axonal elongation design without establishing any contact with each other on DIV 14 (Fig. 2b), indicating that axons from each side grew through the tunnels to the neighboring compartment and could be investigated separately. In the circular tripartite design, axonal extensions were visible through microtunnels on DIV 10, and thicker bundles of axons were observable in the same location in front of the microtunnels on DIV 52 (Fig. 2d).

The development of action potential activity in the circular tripartite network is presented as general spike firing frequencies (Fig. 2e) and in representative raster plots for separate compartments for DIV 10, 24, 38 and 52 (Fig. 2f). The development of neuronal activity followed similar trends as in conventional cultures. The same 3-compartment chip design was previously proven to be successful in preventing fluidic diffusion between compartments^31^, allowing accurate targeted exposure of the proximal compartment to PFF. Fluidic isolation was also confirmed with the application of TTX, as neuronal activity was mainly silenced in the applied (proximal) compartment but preserved or less affected in distal compartments (Supplementary Fig. 3b).

### PFF seeding induces progressive α-syn aggregation and propagation of aggregated forms in a time-dependent manner

**Fig. 3:**
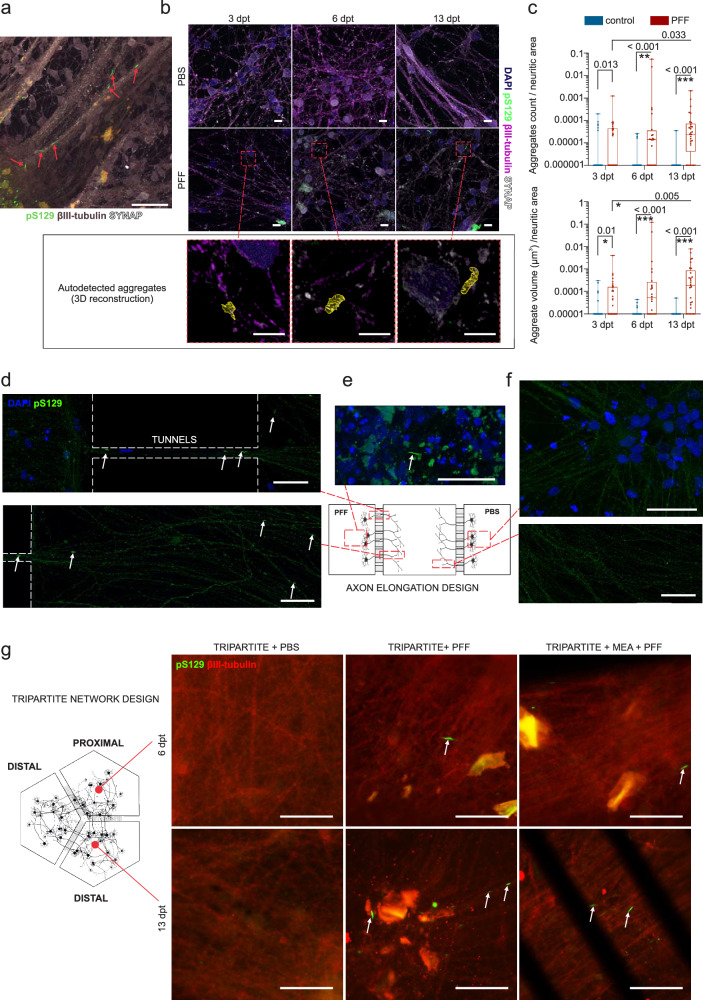
PFF seeded α-s aggregation and propagation of aggregated forms in axon alignment and circular tripartite models. **a** Phosphorylated α-s at residue S129 (pS129, green) localized within aggregates form dense serpentine-like structures, primarily overlap with neuritic markers (βIII-tubulin, purple taupe). **b** Representative images depict PFF treated and control cultures at 3, 6, and 13 dpt with autodetected and 3D-reconstructed α-s aggregates. **c** The number and total volume of detected aggregates per 1 µm^2^ neuritic area are presented as dots and boxplots. Whiskers and horizontal lines indicate the range and the median values, respectively. The number of tiles (*n*) used for quantification ranged from 24 to 36 per group in different time points, with *p* values calculated with Kruskal-Wallis test and significance is adjusted with Bonferroni correction for multiple comparisons (*p < 0.01, **p < 0.002 and ***p < 0.0002). Representative images from the axon alignment model demonstrate pS129-positive α-s aggregates (green, indicated by white arrows) **d** in microtunnels between the PFF-treated and middle compartments, in middle compartment, and **e** within the PFF-treated compartment itself. In contrast, **f** the PBS treated compartment and the middle compartment close to that do not show aggregates. **g** Representative images from the circular tripartite model (with and without MEAs) reveal pS129 positive aggregates (green, indicated by white arrows) on axons (βIII-tubulin, red) in proximal compartment at 6 dpt and distal compartment at 13 dpt. Scale bars in panel **b** represent 5 µm while those in other panels represent 50 µm. The illustrations were generated using Biorender.com.

Conventional and engineered neuronal cultures were treated with a particular PFF strain that was successfully used in previous studies to induce aggregation^32–35^. PFF-induced α-s aggregation was tracked by immunocytochemistry (ICC) by labeling the recruited/aggregated forms of α-s with their phosphorylation at residue S129 (pS129). Aggregates were mainly observed as dense serpentine-like assemblies (Fig. 3a) mostly overlapping with βIII-tubulin or parallel to the extension direction of neuritic bundles in the case of dense neuritic formations (Supplementary video 1). Progression in α-s aggregation was quantified by autodetecting the aggregated forms from 3 days to 13 days post PFF treatment (dpt) in conventional cultures (Fig. 3b). The results indicated an increase both in the number of detectable aggregates and the volumes of the aggregates (Fig. 3c) in the neuronal cultures from 3 dpt to 13 dpt. Thus, we stated these time points in the text as the early (3 dpt), middle (6 dpt) and late phases (13 dpt) of PFF-seeded α-s aggregation.

The propagation of α-s aggregation was initially studied and tracked in the axonal elongation design. Clear phosphorylated serpentine-like assemblies were observable in the PFF-applied compartment at 10 dpt (Fig. 3e). Additionally, phosphorylated assemblies were detected in the axons extending from the PFF-treated compartment to the middle compartment through tunnels as well as in the middle compartment close to the PFF-treated compartment (Fig. 3d and Supplementary Fig. 4a). The control compartment or axons extending from it did not show any similar aggregation patterns (Fig. 3f). Aggregation and anterograde propagation of aggregated forms via the axonal route were observed for all the PFF concentrations tested (5, 10 and 15 µg/ml, Supplementary Fig. 4b). This design clearly showed that anterograde transport is possible, as previously mentioned^36^ and shown^12,14,37^ in the defined timeline of our experimental design.

In the circular tripartite design in which we performed the main functional readouts, aggregation in proximal compartments was not observable in the early phase (3 dpt) but was observable in the middle phase (6 dpt) and propagated to distal compartments in the late phase (13 dpt) (Fig. 3g and Supplementary Fig. 2c). PBS controls did not show any similar aggregation patterns (Fig. 3g).

### PFF-seeded α-s aggregation did not affect cell viability but reduced presynaptic protein content

**Fig. 4:**
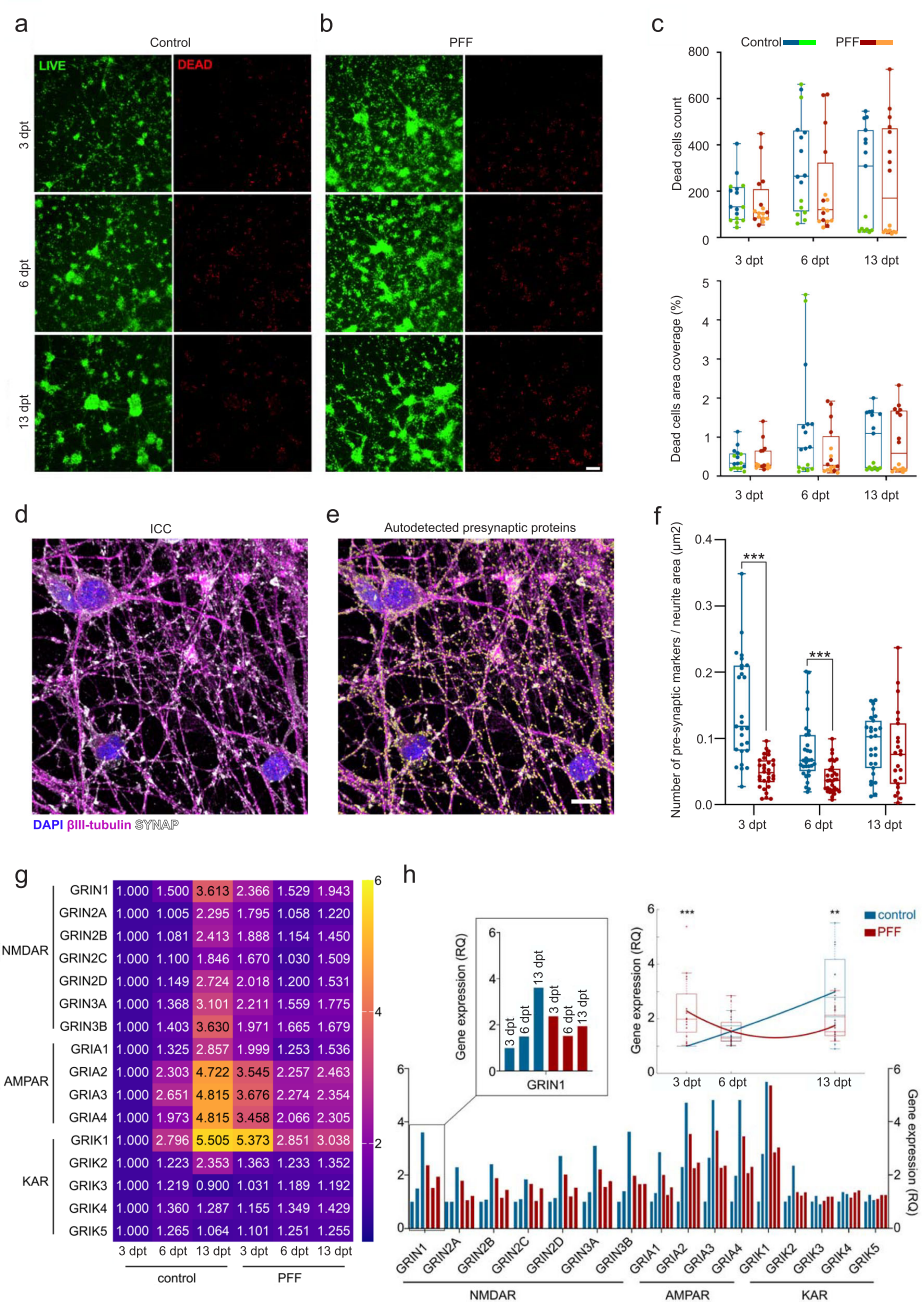
Impact of PFF-seeded α-s aggregation on cell viability, synaptic protein density and iGluR expression. Cell viability and PFF-induced cytotoxicity are evaluated with calcein AM (green) and EtHD-1 (red) live staining for **a** PBS control and **b** PFF treatments at 3, 6, and 13 dpt. Scale bar represents 100 µm. **c** EtHD-1-positive dead cell counts and coverage percentage in PFF-treated vs. control cultures are shown as dots and boxplots over time. Mann-Whitney U test found no significant difference between groups (*p* > 0.05). **d** Representative immunostaining of synaptic proteins and axons, and **e** their autodetection, displaying axons (βIII-tubulin, purple), SYNAP (synaptophysin and synapsin1 together, white), and DAPI (blue); yellow spots indicate autodetected synaptic proteins. Scale bar indicates 10 µm. **f** Synaptic density across days post-treatment is shown as dots and boxplots. Analyzed number of images (*n*) for specified conditions and time points range from 23 to 33 per group in different time points. *p* values calculated with the Mann-Whitney U test (***p < 0.001). Whiskers depict range; horizontal lines show median values. **g** RTqPCR data is displayed with heatmap of RQ values as fold change relative to 3 dpt controls. **h** Same values are shown in columns and as distributions for each time point (*n* = 16) with polynomial fit. Whiskers represent interquartile ranges; horizontal lines indicate median values. Green-orange and blue-red color combinations in panel **c** denote control-treatment pairs in the first and second experiments, respectively. *p* values were calculated with the Mann-Whitney U test (**p < 0.01 and ***p < 0.001).

The impact of PFF seeding on cell viability was investigated with live cell imaging of calcein-AM together with ethidium homodimer-1 (EthD-1). Cell viability was similar for both groups during the experimental timeline, indicating that PFF application was not cytotoxic and did not significantly alter the number of dead cells (Fig. 4a–c) during the experimental timeline compared to controls. Despite the variation in the dead cell count between experiments at 13 dpt, the similarity between the control and PFF-treated groups in each experiment remained consistent. However, some alterations were noted in the βIII-tubulin average pixel intensity inside the calculated neuritic areas at 3 and 6 dpt (Supplementary Fig. 5c) and neurite branching at 6 dpt (Supplementary Fig. 5d).

As down- and upregulation of synaptic proteins^38^ and their reduced colocalization^39^ were previously reported, we assessed the quantities of the presynaptic protein SYNAP during the timeline of the aggregation process (Fig. 4d). Quantification of SYNAP content was performed by automatically detecting the presynaptic *Spots* and associating them with neuritic *Surfaces* (Fig. 4e). The results indicated a significant decrease (*p* < 0.001) in the amount of SYNAP content per unit neuritic area in the early and middle phases of aggregation (Fig. 4f) in PFF-treated cultures, where higher variability was observed in the imaged tiles of the control cultures during the same phases. In the late phase, no significant changes were observed.

We also performed a single-time exploratory analysis to determine the effects of PFF seeding on the gene level expression of ionotropic glutamatergic receptor (iGluR) subunits (Supplementary Table 1). Similar relative expression trends of AMPAR, NMDAR, and the KAR subunits GRIK1 and GRIK2 were found over time within each of the PFF-treated cultures and the control cultures (Fig. 4g). However, the trends between the PFF-treated and control groups were distinct and could be fitted into separate polynomial curves, as depicted in Fig. 4h. These changes in the trends between the control and PFF-treated groups led to significant differences in the relative expression of subunits in PFF-treated neurons compared to the control group, indicating higher overall iGluR expression at 3 dpt (*p* < 0.001, Fig. 4h) and lower overall iGluR expression at 13 dpt (*p* < 0.01, Fig. 4h). No significant differences in expression were observed at 6 dpt between PFF-treated and control cultures.

### PFF-seeded α-s aggregation reduced calcium activity and mitochondrial motility during different phases of α-s aggregation

The temporal impacts of PFF-seeded α-s aggregation on neuronal functioning were investigated in conventional cultures by first analyzing calcium transients/oscillations in neurons and mitochondrial motility in axons from the early to late phase.

**Fig. 5:**
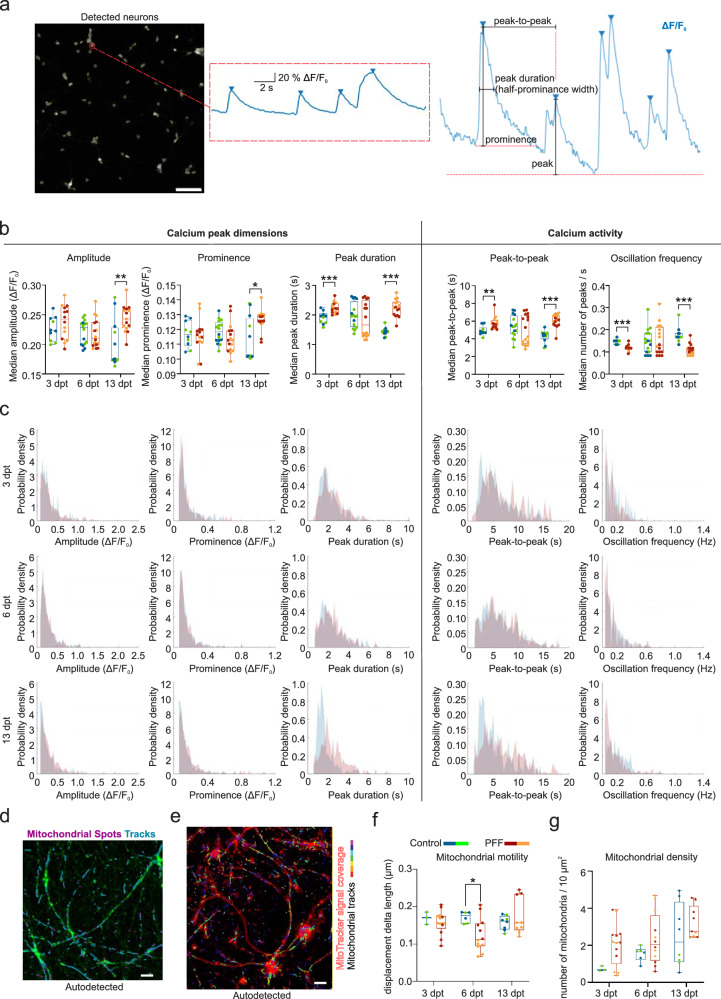
Impact of PFF-seeded α-s aggregation on mitochondrial motility and calcium oscillations. **a** Representative auto-detected neuron region showing calcium oscillations as relative percent fluorescence change from baseline. Scale bar is 100 µm. Parameters derived from calcium oscillations are displayed. **b** Calcium peak dimension parameters (amplitude, prominence, and peak duration) and calcium activity parameters (peak-to-peak and oscillation frequency) are quantified. Data points represented the median values of the neurons in each recording. The number of recordings (*n*) used for quantification range from 10 to 16 per group in different time points. **c** Histograms represent distributions of values obtained from each neuron in the recording regions shown in (**b)**. The number of neurons (*n*) included in recordings range from 475 to 1465 per group in different time points. **d** Mitochondrial motility and density were assessed using MitoTracker live staining (green) by detecting mitochondrial spots (purple) and tracks (turquoise) automatically. Scale bar is 5 µm. **e** MitoTracker signal coverage area is indicated with red lines. Colors on mitochondrial track represent different time frames. Scale bar is 5 µm. **f** Mitochondrial motility is quantified by means of displacement delta length and **g** Mitochondrial density is quantified by number of mitochondria per 10 µm^2^ area. Whiskers and horizontal lines in quantifications indicate the range and the median values, respectively. Green-orange and blue-red color combinations denote control-treatment pairs in the first and second experiments, respectively. *p* values were calculated with the Mann-Whitney U test to compare groups at each time point (*p < 0.05, **p < 0.01 and ***p < 0.001).

Calcium oscillations were studied via changes in calcium binding fluorescence intensity levels in the automatically detected neurons (Supplementary video 3). Figure 5a shows the automatically detected neuronal regions in Supplementary video 3 and calcium oscillations from the labeled neuron as well as the parameters derived from calcium oscillations. Calcium oscillations were assessed by means of peak dimension parameters (amplitude, prominence, and peak duration) and calcium activity parameters (peak-to-peak and oscillation frequency, Fig. 5b). A significant decrease in the calcium oscillation frequency and an increase in the peak-to-peak and peak duration of calcium waves were observed at 3 and 13 dpt (Fig. 5b). Additionally, calcium oscillations had higher peaks in the PFF-treated group (amplitude, *p* < 0.01 and prominence, *p* < 0.05) at 13 dpt (Fig. 5b). Normalized histograms (Fig. 5c) represent these changes for all the detected neurons from the same regions analyzed in Fig. 5b. Changes in the distribution of peak dimension and calcium activity parameters for the PFF treatment and control groups at 3 and 13 dpt were also evident in histograms (Fig. 5c). Median values of recordings showed experimental variation for some analyzed parameters at 6 dpt; however, the similarity between the control and PFF-treated groups within each experiment remained mostly consistent (Fig. 5b), which can be also observed from the histograms (Fig. 5c).

Mitochondrial trafficking was evaluated via motility and represented by displacement of mitochondria in the mitochondrial tracks (Fig. 5d, e and Supplementary video 2). Mitochondria and tracks were automatically detected from recorded live time-lapse images of 90 s (Fig. 5d, e), and mitochondrial displacement in the tracks was calculated from those images. While experimental variations were observed, mitochondrial motility (displacement delta length) decreased significantly at 6 dpt compared to the control (*p* < 0.05, Fig. 5f). No significant change was noted for the number of motile mitochondria (Fig. 5g).

### PFF-treated circular tripartite networks reveal a reduction in neuronal activity and depict distinct responses to glutamatergic modulators

**Fig. 6:**
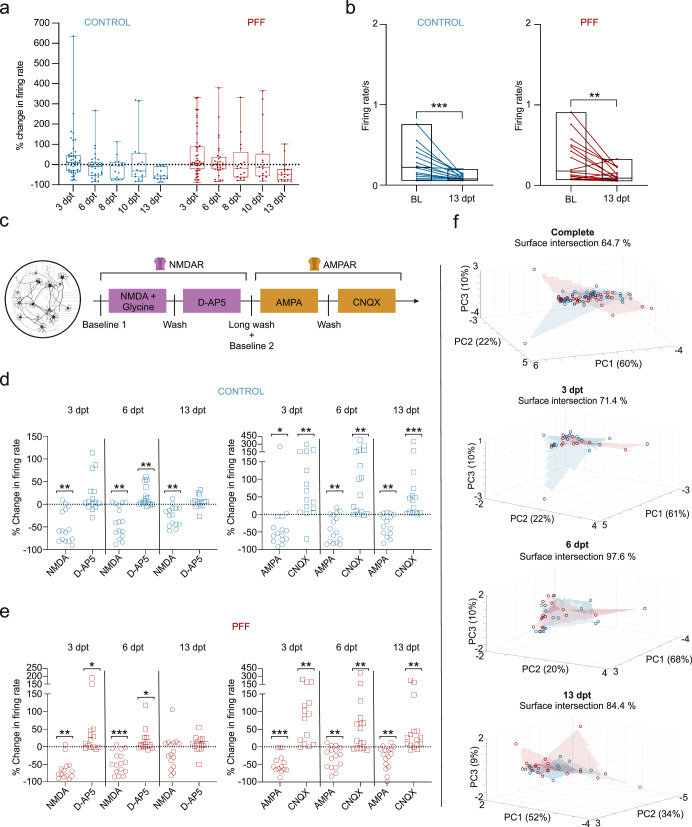
Spontaneous and glutamatergic modulator induced neuronal activity in conventional networks after PFF treatment. **a** Illustration of the percent changes in neuronal activity, with the dotted line representing baseline activity recorded prior to PFF treatment. **b** Pairwise analysis depicting mean firing rates at 13 dpt compared to baseline values recorded in the same well before PFF or PBS vehicle treatment. **c** A schematic representing serial stimulation with glutamatergic modulators, namely, NMDA in conjunction with Glycine, D-AP5, AMPA, and CNQX. **d** Outputs to each glutamatergic modulator were presented as percent changes to the previously calculated neuronal activity in the same well (dotted lines) for controls and **e** for PFF treated networks. **f** 3D representation of multiparametric analysis presented with the first three principal components (PC1-3) and calculated intersection percentages between the surfaces of control and PFF treated network values. Each data point in the images represented values obtained from a conventional MEA well. The number of MEA wells (*n*) included to analysis range from 14 to 16 per group in different time points. Whiskers and horizontal lines in quantifications indicate the range and the median values, respectively. p values were calculated with the Wilcoxon Signed Ranked test for pairwise comparisons to baseline recordings (**b**) or to conditions prior to stimulations (**d**, **e**) (*p < 0.05, **p < 0.01 and ***p < 0.001). The illustrations were created using Biorender.com.

**Fig. 7:**
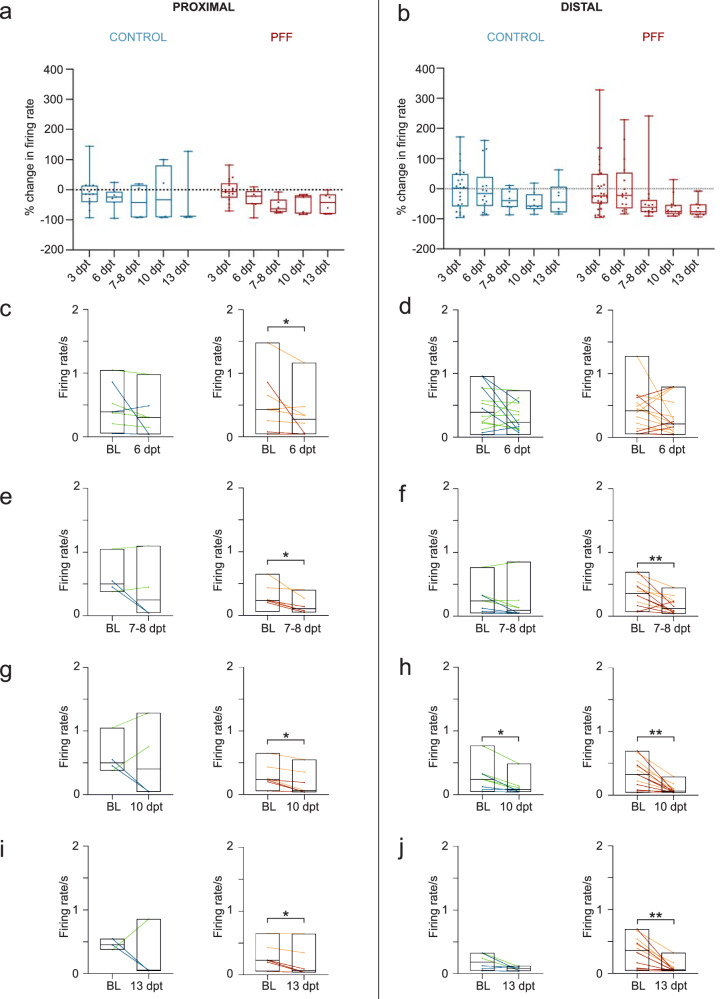
Spontaneous neuronal activity in circular tripartite networks after PFF treatment. The percent changes in neuronal activity relative to baseline activity recorded in the same network prior to PFF treatment (dotted line) **a** for proximal compartment and **b** for distal compartments. **c**, **e**, **g**, **i** Pairwise analysis of mean firing rates relative to baseline values recorded in the same network prior to PFF treatment at different dpt for proximal compartments, and **d**, **f**, **h**, **j** for distal compartments. Each data point in the images corresponds to values obtained from an individual circular tripartite network. The number of circular tripartite networks included to analysis for proximal compartments is *n* = 3–13 for control and *n* = 6–17 for PFF treated, while for distal compartments are *n* = 6–25 for control and *n* = 12–33 for PFF treated. Green-orange and blue-red color combinations denote control-treatment pairs in the first and second experiments, respectively. Box plots and horizontal lines in quantifications indicate the range and the median values, respectively. *p* values were calculated with the Wilcoxon Signed Ranked test for pairwise comparisons (*p < 0.05 and **p < 0.01).

The changes in neuronal activity were assessed in both conventional and circular tripartite networks from the early to late phase of α-s aggregation. In conventional cultures, the percent changes in neuronal activity relative to baseline activity before PFF treatment were similar for both PFF-treated and control networks (Fig. 6a). Pairwise analysis of firing rates revealed a significant reduction in neuronal activity at 13 dpt for both treated and control cultures (Fig. 6b). On the other hand, circular tripartite networks exhibited a more pronounced decrease in neuronal activity in PFF-treated cultures when recordings from proximal and distal compartments were assessed separately (Fig. 7a, b). The percent changes in neuronal activity to baseline activity before PFF treatment showed a slight difference in both proximal (Fig. 7a) and distal (Fig. 7b) compartments between PFF-treated and control networks. However, the nuance between PFF-treated and control networks was revealed by comparing the activity to their own baselines. The firing rate of PFF-treated networks significantly decreased starting from 6 dpt in the proximal compartment (Fig. 7c, *p* < 0.05) and 7-8 dpt in the distal compartments (Fig. 7f, *p* < 0.01) and was consistently observed up to 13 dpt (Fig. 7e-j).

**Fig. 8:**
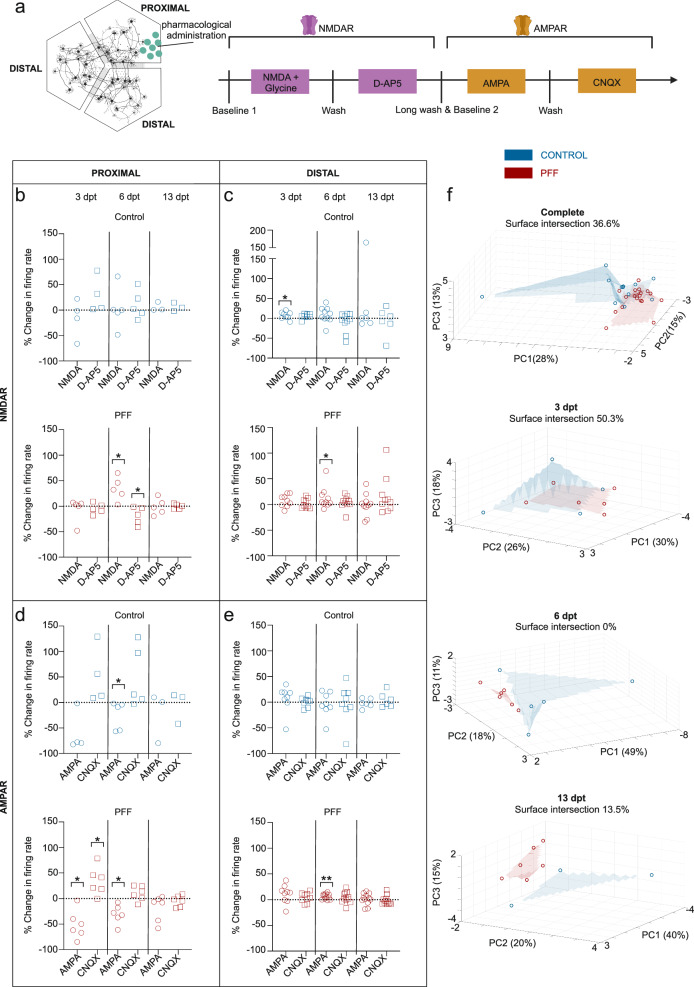
Glutamatergic modulator induced neuronal activity in conventional networks after PFF treatment. **a** Schematic for the serial stimulation with glutamatergic modulators, NMDA together with Glycine, D-AP5, AMPA and CNQX which is applied to the proximal compartment. **b**–**e** Outputs to each glutamatergic modulator were presented as percent changes to the previously calculated neuronal activity in the same network (dotted lines) for NMDAR modulators **b** in proximal and **c** distal compartments as well AMPAR modulators **d** for proximal and **e** for distal compartments. **f** 3D representation of multiparametric analysis presented with the first three principal components (PC1-3) and calculated intersection percentages between the surfaces of control and PFF treated circular tripartite network values. Each data point in the images represented values obtained from a circular tripartite network. The number of networks included to NMDAR modulator analysis for proximal compartments is *n* = 3-5 for control and *n* = 5 for PFF treated, while for distal compartments *n* = 6–10 for control and *n* = 9-10 for PFF treated. The number of networks included to AMPAR modulator analysis for proximal compartments is *n* = 3-5 for control and *n* = 6 for PFF treated, while for distal compartments *n* = 6–8 for control and *n* = 9–12 for PFF treated. *p* values were calculated with the Wilcoxon Signed Ranked test for pairwise comparisons (*p < 0.05 and **p < 0.01). The illustrations were created using Biorender.com.

The responses of neuronal populations to serial stimulation with the glutamatergic modulators NMDA, glycine, D-AP5, AMPA and CNQX were investigated in both conventional and circular tripartite networks (Figs. 6c and 8a). The stimulations were performed at 3, 6, and 13 dpt. Outputs to each glutamatergic modulator are presented as percent changes from the previously calculated neuronal activity to observe the conditional effect (Fig. 6d–e and Fig. 8b–e). Absolute firing rates for proximal compartments present the same trends in different view (Supplementary Fig. 6) and baseline mean firing rates prior to stimulations are similar between groups (Supplementary Fig. 7a).

In conventional cultures, the responses to glutamatergic modulators were similar for the control (Fig. 6d) and PFF-treated groups (Fig. 6e). There was a significant decrease in neuronal activity with NMDA + glycine and AMPA exposure, followed by a significant increase with D-AP5 and CNQX exposure, respectively (Fig. 6d, e), in conventional networks. The exception was at the 13 dpt NMDA + Glycine and D-AP5 application, where a similar trend could still be observed (Fig. 6d, e). A multiparametric assessment was conducted to investigate whether the responses of the PFF-treated and control networks to the modulators could be separated (see Figs. 6f and 8f). The results of the multiparametric analysis are represented using the values of the first three principal components (PCs). Analysis showed a high overlap between the control and treatment groups in conventional networks, with overlapping percentages of 71.4%, 97.6%, and 84.4% for 3, 6, and 13 dpt, respectively (Fig. 6f). Assessment of the pooled data from all three time points (64.7% overlap between the two groups, Fig. 6f) and overlapping percentages from individual time points indicated that the responses of the treatment and control groups in conventional networks did not form easily distinguishable clusters (Fig. 6f).

In the circular tripartite network, serial stimulations were performed in the proximal compartment (Fig. 8a), and responses were analyzed for both proximal and distal compartments (Fig. 8b–e). The responses to glutamatergic modulators differed from those observed in conventional cultures (Fig. 8b–e). In the proximal compartment, NMDA + glycine and subsequent D-AP5 exposure to PFF-treated networks at 6 dpt elicited an opposite response compared to PFF-treated cultures in conventional cultures at 6 dpt (c.f. Fig. 6e and Fig. 8b). AMPA and following CNQX exposure on PFF-treated cultures showed a similar response trend in the proximal compartments of the circular tripartite network as observed in conventional cultures at 3 dpt (c.f. Fig. 6e and Fig. 8d). However, the responses for other time points varied and did not follow a consistent trend. Multiparametric analysis showed that at 6 and 13 dpt, the responses of the control and PFF-treated circular tripartite networks were easily distinguishable from each other, with overlapping percentages of 0% and 13.5%, respectively (Fig. 8f). When considering the pooled data from all three time points, there was a 36.6% overlap between the two groups (Fig. 8f).

Altogether, the findings demonstrated that the sequential modulation of glutamatergic receptors produced mixed effects. However, distinction between control and PFF-treated networks became evident through a comprehensive multiparametric evaluation of the proximal and distal subpopulations within the circular tripartite network.

## Discussion

In this work, functional alterations during α-s aggregation and propagation in hiPSC-derived cortical networks over time were studied in detail. To enhance our investigation, we utilized innovative microfluidics culturing techniques with integrated MEAs in addition to conventional cultures, allowing us to conduct spatiotemporal analysis of α-s aggregation and propagation in interconnected neuronal networks. The impacts of PFF-seeded α-s aggregation were examined at the gene expression level, cellular level (presynaptic protein content, calcium and mitochondrial functionality), and population level (neuronal activity) during the early, middle, and late stages of a 13-day follow-up period. The analysis revealed a multifaceted relationship between structural and functional changes from the cellular to population level during the in vitro propagation of α-s aggregates, underscoring the importance of functional assessment in a timeline process for evaluating the phases of α-s aggregation.

hiPSC-derived neuronal cells are widely used to investigate a variety of late-onset disorders to demonstrate the expression of disease phenotypes (reviewed in refs. ^40,41^), as reported for modeling α-s aggregation and its effects^14,23,42^. To study α-s aggregation and propagation, it is important that neuronal models contain synapses where α-s are naturally localized and that neurons are functional. Some studies have investigated PFF seeding on primary rodent neurons before DIV 14 and have found that α-s aggregation induced by PFF is observable in this early stage^13,21^, despite neurons generally lacking mature synapses at this point^43^. However, they observed an increased efficiency of PFF seeding as the neurons matured, accompanied by higher expression of α-s at the presynaptic terminal, highlighting the importance of considering the developmental stage of cultures during experiments^13^. Here, we characterized the maturity of human cortical neurons by means of progressive α-s expression and their localization in the presynapses; we followed the synaptic development process from DIV 7 to DIV 49. The continuation of synaptic formation was also taken into consideration in the experimental setup, as it could distort the influence of α-s aggregation on network functionality. Thus, PFF seeding experiments were started when the network no longer showed an increasing trend in network firing, from DIV 49 onwards. Previous studies with human neurons have used cultures over DIV 30^14,23^, settling quite well to the same developmental stage as in our study. The reach in activity “peak” followed by a decrease is typical for hiPSC-derived neurons^44^, and in line with this, the activity dropped at a later stage, but neurons were still functional as assessed with MEA recordings and calcium imaging.

The engineered cultures in in-house developed microfluidic chips^29–31^ enabled elongation of axons from isolated soma compartments, allowing us to demonstrate progressive α-s aggregation and axonal propagation after PFF treatment in human neurons. Previously, the same PFF strain was used to induce similar effects in rodent neurons^32–35^. The transportation of α-s from proximal to distal compartments occurred in a time-dependent manner, from Day 6 onward after PFF treatment, in a similar manner as shown in previous studies^13,14^. PFF-initiated alpha-synuclein aggregation did not affect cell viability, which is in line with previous works performed with different PFF strains applied at comparable concentrations^11,39^. Last, as with the tripartite model containing both microfluidics and integrated MEA, we have shown that the interconnected neuronal networks form functional connections^31^. We consider our setups used here relevant for a comprehensive, temporal and multifaceted approach to study aggregation and propagation.

Temporal analysis of presynaptic protein quantity and iGluR subunit expression was performed in conventional cultures. Previously, both downregulation and upregulation of various synaptic proteins have been reported during the development of α-s pathology^38^; however, these analyses lacked temporal assessment. For example, studies utilizing a PFF-seeded a-s aggregation model with human^42^ and mouse neurons^13^ found no changes in synaptophysin quantity at 14 dpt, which aligns with our late, 13 dpt, phase result where no changes in synaptophysin and synapsin 1 quantity were observed. Interestingly, we found that their quantity was decreased at the early (3 dtp) and mid (6 dpt) phases of α-s aggregation, which was not examined in previous works^13,42^. However, as a reduction in the colocalization of presynaptic Synapsin 1 and postsynaptic PSD95 proteins in primary mouse neurons at 7 dpt was reported^39^, in line with our results, synaptic content reduction following neuronal activity alterations could occur at earlier phases of α-s aggregation and propagation.

We also investigated the timeline alterations in the gene expression of iGluR subunits during α-s aggregation. Our single-time exploratory temporal analysis provided a crude overview, particularly on the similarities of the expression of AMPAR and NMDAR subunits within control and PFF-treated cultures. A significant alteration in the regulation of gene level expressions of these receptor subtypes was evident in both the early and the late phases of PFF treatment. However, additional assessment is required to validate if these alterations would be reflected in protein level. Previously, decreases in levels of GluN2A (gene: GRIN2A)- and GluN2B (gene: GRIN2B)-containing NMDA receptors were reported in rat hippocampal neurons after PFF treatment in vitro and in vivo^22,45^, and reorganization of these receptors has been shown after PFF application, which impacts neuronal activity^25^. There is limited research on KAR subunits in comparison to NMDAR and AMPAR subunits^46^; however, our study highlights the GRIK1 subunit as having the highest changes during the process, warranting further investigation.

Calcium signaling and mitochondrial functionality were assessed during progressive α-s aggregation in conventional cultures. Our results indicated an increase in the dimension of calcium peaks and a reduction in their oscillation frequency. Reduced oscillation frequency^14,21,22^, along with alterations in peak characteristics such as increased peak duration or changes in amplitude^14,21^, have been observed in association with α-s aggregation at different time points. While Gribaudo et al. presented findings on calcium signaling in hiPS-derived cortical neurons at 1 and 19 dpt^14^, Froula et al. ^21^ and Huang et al. ^22^ focused on rodent hippocampal neurons at 4 and 7 dpt, all of which are indicative of neuronal dysfunction. Interestingly, we detected these changes in the early and late phases of aggregation, which is in line with the only comparable study available showing changes in the same parameters in the early and late phases (c.f. 1 and 19 dpt) after exposure of hiPSC-derived cortical cells to human recombinant α-s PFFs^14^. This indicates that mechanisms behind calcium behavior are dynamic in nature and that potential compensatory mechanisms may take place at the middle phase.

The effect of developing α-s pathology on mitochondrial dynamics was assessed by means of mitochondrial motility and quantity. Although our results suggested the loss of mitochondrial functionality, we did not see a significant change in mitochondrial density. The reduction in mitochondrial trafficking and arrest before axonal degeneration has been reported in different PD neuronal models^47^. The possible mechanisms of this relationship were reported in primary mouse neuronal cultures where aggregated forms of α-s induced structural and functional mitochondrial damage at 7 dpt, leading to mitochondrial fission and mitophagy at 14 dpt^48^. To study this temporal relationship more thoroughly, further studies are required. Our model has potential to indicate the critical timelines for investigating these dynamic processes in future studies.

Previously, dynamic interplay between calcium oscillations and mitochondrial motility has been suggested in which frequency-modulated oscillations regulate mitochondrial motility, with maximal motility occurring during cytosolic calcium resting states and complete suppression occurring during calcium spikes and oscillations^49^. It is worth investigating whether our findings stem from the same mechanisms disrupted by aggregated α-s, given that changes in calcium signaling were detected at 3 and 13 dpt, and alterations in mitochondrial functionality were observed at 6 dpt.

Here, MEAs were used to examine extracellular functional responses of neurons during PFF-induced α-s aggregation, allowing longitudinal monitoring of the same networks. Notably, both PFF-treated and untreated cultures exhibited a similar decreasing trend in neuronal activity over the experimental timeline in conventional networks. Previous MEA studies after PFF application in conventional cultures have reported mixed results^11,23–25^. For instance, a decrease in neuronal activity was observed at specific time points (7 and 10 dpt) in primary mouse hippocampal^25^ and rat cortical^11^ networks after exposure to PFFs. In contrast, no functional changes were detected during a 3-week period after PFF treatment in hiPSC-derived neuronal networks^23^ or between 14 and 21 dpt in primary rat enteric neurons^24^. Taken together, conventional MEA seems to be inefficient in capturing changes in neuronal activity during α-s aggregation in hiPSC-derived neurons. Most likely, in a single network MEA setup, changes in neuronal activity after PPF exposure may be masked or averaged out. The efficiency of capturing changes depends on factors such as fibril strain, species origin of the neurons in the network, synaptic development, and culture age. Additionally, these factors can also affect other measured parameters, so they are not necessarily relevant for functional studies.

By achieving successful axonal transfer of α-s aggregates between isolated neuronal subpopulations in a circular tripartite network, the timeline of pathological progression was delineated into three distinct phases. These phases are characterized as follows: an early phase with no observable aggregates in the proximal compartment, a middle phase with observable aggregates exclusively in the proximal compartment, and a late phase with observable aggregates in the proximal compartment and propagated aggregates in the distal compartments. In this context, the circular tripartite design offered the advantage of dissecting spatial information with simultaneous assessment of proximal and distal compartments at the functional level. Notably, we observed a consistent decrease in mean firing rates in both proximal and distal compartments of the PFF-treated cultures in the circular tripartite network. Reduced neuronal activity correlated with the middle and late phases of pathological α-s progression and diverged from controls, which showed more fluctuating dynamics in their proximal and distal compartments.

Moreover, our results indicate that conventional cultures are unable to reveal any differences in responses to iGluR modulators between PFF-treated and control networks. However, by utilizing a circular tripartite design, we detected distinctions, particularly in NMDAR-modulated signaling at 6 dpt. This illustrates that the circular tripartite design is more adept at uncovering changes in the multiscale system’s input/output dynamics^50^. Furthermore, our multiparametric analysis of both proximal and distal subpopulations revealed pronounced distinctions in glutamatergic receptor-modulated neuronal activity during the middle and late stages of α-s aggregation at both 6 and 13 dpt between PFF-treated and untreated networks. Partial separation between the groups were also observed when the data from these two indicative time points were pooled (Supplementary Fig. 7b). These observations suggest a driving factor in glutamatergic signaling, likely influenced by aggregated forms of α-s. In fact, earlier research has highlighted the indirect role of PFF on NMDA-type glutamate receptors, particularly GluN2A, via a synaptic protein called rabphilin-3A at 7 dpt^45^. The acute impact of α-s aggregates on glutamatergic signaling involving the modulation of NMDAR subtype GluN2A at the single-cell level has also been reported^51^. In summary, glutamatergic receptor-mediated neuronal activity, particularly via NMDAR, is influenced by α-s aggregation. This effect is elusive when observing population-level neuronal activity unless a more comprehensive approach is employed to spatiotemporally dissect the information from the applied network.

**Fig. 9:**
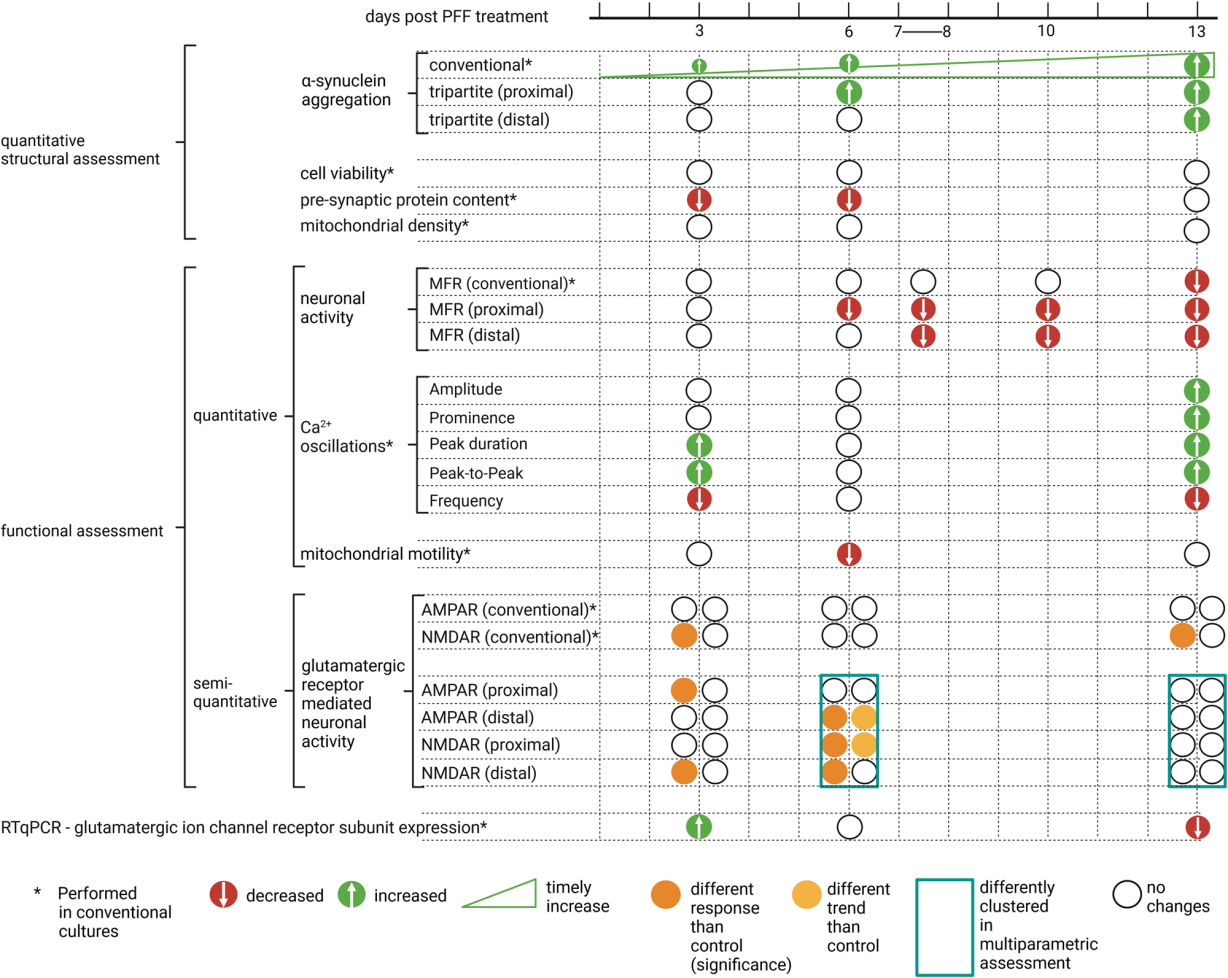
Chronological table of the functional and structural changes that occurred throughout the α-s aggregation process. Timeline of the functional and structural changes in PFF treated networks are presented in relation to their distinctions from control networks. Significant increase and decrease obtained via quantitative assessment were presented green and red circles, respectively. In addition to identifying significant changes, semi-quantitative assessments such as clustering and observed trends are included based on their deviations from controls. Figure was created using Biorender.com.

Together with the proposed circular tripartite network and the conventional network models, a comprehensive assessment was possible. By incorporating the timeline of the findings and different methodologies used, we were able to provide a chronological table of the functional and structural changes that occurred during follow-up (Fig. 9). The changes were assessed both quantitatively and semiquantitatively for the PFF-treated networks in comparison to control networks.

Further exploration is required to determine whether the changes in the quantity of synaptic proteins and iGluRs directly correlate with the timeline of alterations in neuronal activity. The decrease in the presynaptic proteins in the early and middle phases did not simultaneously influence neuronal activity, indicative of more intricate mechanisms at play, where the upregulation or downregulation of various synaptic proteins collectively drive the observed changes in neuronal activity, as reported earlier^38^. A detailed analysis is required to investigate the complex interplay of synaptic proteins during the early phase of α-s aggregation and its association with reorganization and alterations of iGluRs. Outcome of such analysis would be rewarding to understand the underlying mechanisms of altered glutamatergic receptor-mediated neuronal activity. In addition, possible relationships in the patterns of calcium signaling and mitochondrial activity have promise for further investigation, providing valuable insights into the mechanisms underlying the progression of α-s pathology and leading to the exploration of potential therapeutic targets.

In conclusion, we showed that the use of a comprehensive and time-based analysis model is crucial to understand the interplay of various functional changes during the progression of α-s pathology. We employed a nonconventional culturing paradigm in a circular tripartite network with improved analysis approaches to investigate the temporal impact of α-s pathophysiology on neuronal activity in vitro. Our model is suggested as an incremental step to bridge the gap between conventional in vitro and in vivo PD studies; moreover, it enables focusing on human neurons, especially from PD patients, in future studies. The ability of the model to distinguish between different treatment locations and to analyze them over time within the networks being studied shows potential for testing/validating the precision and effectiveness of preventive or therapeutic medications.

## Methods

### Cortical neuronal differentiation and culturing

In this study neuronal cells differentiated from hiPSC line UTA.04511.WTs were used^28^. The hiPSCs were acquired from voluntary subject who had given written and informed consent. The project has supportive statement from Ethics Committee of the Expert Responsibility area of Tampere University Hospital to use the named hPSC line in neuronal research (R20159). hiPSCs were expanded and differentiated into cortical neurons in a feeder-free culture, as previously described^44,52^. The cells were cultured using neural maintenance medium (NMM) as a basal medium consisting of 1:1 DMEM/F12 with Glutamax and Neurobasal, 0.5% N2, 1% B27 with retinoic acid, 0.5 mM GlutaMAX, 0.5% NEAA, 50 µM 2-mercaptoethanol, 0.1% penicillin/streptomycin (all from Thermo Fisher Scientific) and 2.5 µg/ml insulin (Sigma). During the neural induction stage (days 1–12 of differentiation, Supplementary Fig. 1), the NMM was supplemented with the small molecules 100 nM LDN193189 (Sigma) and 10 µM SB431542 (Sigma). For neural proliferation (days 13–25, Supplementary Fig. 1), the NMM was supplemented with 20 ng/ml fibroblast growth factor-2 (FGF2, Thermo Fisher Scientific). For the final maturation (days 26–32, Supplementary Fig. 1), the cells were cultured in NMM containing with 20 ng/ml brain-derived neurotrophic factor (BDNF, R&D Systems), 10 ng/ml glial-derived neurotrophic factor (GDNF, R&D Systems), 500 µM dibutyryl cyclic adenosine monophosphate (db-cAMP, Sigma) and 200 µM L-Ascorbic acid (AA, Sigma). At day 32 (hereafter referred as DIV 0, Supplementary Fig. 1), the cells were plated for experiments a density of 19,000 to 50,000 cells/cm^2^ on Ø 13 mm coverslips, 1 × 10^6^ cells/cm^2^ on Axion well-plate MEAs (Axion BioSystems, Atlanta, GA, USA) or 286,000–333,000 cells/cm^2^ on microfluidics devices^29,31^ in NMM supplemented with 10 µM ROCK inhibitor (Sigma). Coverslips were coated with 12 µg/cm^2^ poly-L-ornithine (PLO, Sigma) and 15 µg/mL LN521 (Biolamina). Microfluidics devices and MEAs were coated with 0.1% poly-ethylenimine (PEI, Sigma) and 50 µg/ml LN521. Prior to coating and cell plating, the microfluidics devices were treated with oxygen plasma in a Pico plasma system (4 min at 50W; Diener electronic GmbH + Co. KG, Ebhausen, Germany) for sterilization and to make them hydrophilic. The next day (DIV1), NMM was replaced with BrainPhys™ Neuronal Medium (Stemcell Technologies) for the rest of the experiment supplemented with 0.5% N2, 1% B27 and 0.1% Penicillin-Streptomycin. The samples were cultured under standard incubator conditions (+ 37 °C, 5% CO2) and the medium was changed three times per week.

### Engineered neuronal cultures

An engineered cortical model was created with two designs: axonal elongation^29^ (Fig. 2a) and circular tripartite^31^ (Fig. 2c). Both designs have tunnel architecture with the same width and height between cell compartments (Fig. 2a, c), providing a more accurate in vitro cortical model for α–s propagation by isolating the somatic compartments and enabling spatial separation of treatment regions. The network architecture and chip dimensions are shown in Fig. 2a, c. Here, the axonal elongation design was used for testing and optimizing PFF seeding protocols after DIV 14 (Supplementary Fig. 1b), whereas the circular tripartite design was used for functional experiments after DIV 49/56 (Supplementary Fig. 1a).

In the axonal elongation design, neurons were seeded to the side compartments, and axons from these populations extended toward the middle compartment from opposite ends (Fig. 2a). Thus, in one chip, one side compartment was used for PFF treatment and the other as a PBS control. α–s propagation was tracked from corresponding locations in the middle compartment.

In the circular tripartite design, neurons were seeded into all three compartments, forming an intranetwork freely as well as an intercompartmental network via microtunnels, thus establishing a circular network with three separate subpopulations (Fig. 2c). In this design, one compartment was exposed to aggregated α-s during the treatment (proximal), and two distal networks were vulnerable to pathological strains only via structural connections with the proximal network (Fig. 2c). MEAs are also embedded in a circular tripartite design to collect functional readouts during the process.

The circular tripartite design was utilized for the main functional analysis to follow neuronal activity. *n* = 32 MEA chips with the circular tripartite design were utilized throughout the study. The number of chips was decreased after each endpoint recording, which was performed at 3, 6 and 13dpt. The details of the number of chips used are provided in Supplementary Table 2.

### Immunocytochemical stainings

The expression of neuronal markers and soluble and aggregated forms of endogenous α-s were investigated with ICC. The neuronal samples on Ø 13 mm glass coverslips were stained with similar protocol as described previously^53^. The neuronal samples in the circular tripartite and axonal elongation devices were stained utilizing the same staining protocol with some modifications as described previously^29^. Primary antibodies consisted of βIII-tubulin (chicken, 1:200 for conventional cultures and 1:50 for cultures on microfluidics, Abcam, ab41489), βIII-tubulin (mouse, 1:1000 for conventional cultures and 1:500 for cultures on microfluidics, Sigma, T8660), MAP-2 (chicken, 1:4000 for conventional cultures and 1:2000 for the cultures on microfluidics, Novus, NB300-213), SYNAP is a cocktail of Synaptophysin and Synapsin 1 as they are used followingly: Synaptophysin (mouse, 1:500 for both conventional cultures and cultures on microfluidics, Abcam, ab8049), Synapsin 1 (mouse, 1:500 for both conventional cultures and cultures on microfluidics, Invitrogen, MA5-31919). Alpha-synuclein (rabbit, 1:2000 for conventional cultures, Abcam, ab138501), Phospho-α-Synuclein (pS129, D1R1R) (rabbit, 1:1000-2000, for conventional cultures and 1:1000 for cultures on microfluidics, Cell Signalling Technology, 23706 S), vGlut1 (rabbit, 1:2000 for conventional cultures, Synaptic Systems, 135303) and PSD-95 (mouse, 1:50 for conventional cultures, Abcam, ab2723). Secondary antibodies consisted of Alexa Fluor 488 (1:400), Alexa Fluor 568 (1:400) or Alexa Fluor 647 (1:200) dyes (all Thermo Fisher Scientific). Secondary antibodies were used at higher concentration for microfluidics Alexa Fluor 488 (1:200), Alexa Fluor 568 (1:200) and Alexa Fluor 647 (1:125). The cells were imaged using an Olympus IX51 microscope with an Olympus DP30BW camera (Olympus Corporation, Hamburg, Germany), an LSM780 laser scanning confocal microscope with a Quasar spectral GaAsP detector (all from Carl Zeiss, Jena, Germany).

### Western blots

Endogenously expressed α-s in hiPSC-derived neurons was examined during DIV 14 to 28. Neurons were lysed 14 and 28 days after plated in 6-well plates with Triton X supplemented with 1% protease and phosphatase inhibitors (all from bimake.com). Lysates were centrifuged for 30 min at 14000 rpm at 4 °C to eliminate cell debris and supernatant fractions were collected. Protein concentration was determined by using Pierce 660 nm Protein Assay (Thermo Fisher Scientific). 9 µg of protein and page ruler (Thermo Fisher Scientific) were loaded on an 8–16% SDS-polyacrylamide gel and transferred to nitrocellulose membrane (all from BioRad). Electrophoresis was run for 10–15 min at 80 V and then for 45 min at 120 V. After the membranes were blocked for 1 h with 4% bovine serum albumin (Sigma), they were incubated with primary antibodies α-s (rabbit, 1:2000, Abcam, ab138501) and β-actin (mouse, 1:2000, Santa Cruz Technologies, sc-47778) overnight at 4 °C, and then the membranes were washed and incubated with secondary antibodies (rabbit, 1:10000, Li-Cor, 32213 and mouse, 1:10000, Li-Cor, 32212) for an hour at room temperature. Images are acquired via Image Studio v5.2 (Li-Cor Biosciences) and quantified in ImageJ.

### S129A α–s PFF seeding

Human recombinant α-s PFFs that were used in this work consisted of S129A-mutant α-s. Since pathological α-s aggregates are primarily phosphorylated at S129^2^, we utilized fibrils of mutant α-s that cannot be phosphorylated at this site. This ensured labeling of merely endogenously phosphorylated α-s while avoiding the externally added S129A PFFs. PFFs were produced at Aarhus University. The generation, validation and usage information of PFFs was previously described in several studies^32–35^.

After thawing the PFFs (stock concentration was 2 mg/mL in PBS), they were diluted in cell culture medium (NMM or BrainPhys™ Neuronal Medium) to obtain the working concentration. The prepared PFF solution was sonicated with a Vibra Cell Sonicator (Sonics & Materials Inc., Newtown, USA) with a 100 ms pulse on and off and 20% amplitude for 1–1.5 min to ensure homogeneity and the size of the PFFs prior to addition to cell cultures.

For the conventional cultures, the application of PFF and PBS vehicle solutions was conducted at DIV 49 or 56 by removing the culture media and applying 150 µl or 200 µl of the treatment solution on 48-well plates and 24-well plates, respectively. After 24 h, 350 µl or 800 µl of cell culture media was added on top of the treatment solution for a total final volume of 500 µl or 1 ml, respectively. For testing and optimizing α-s aggregation and propagation via axonal transport, 100 µl of PFF solution was added to one of the side compartments of the axonal elongation model at DIV14 at a final concentration of 5, 10 or 15 μg/ml. Before PFF addition, all the media from the top medium reservoirs^29,31^ and 10 µl from the bottom cell chambers were removed. For the other side compartment, PBS in NMM solution was added with the same protocol as a vehicle control. After 24 h of treatment, the PFF and PBS solutions were washed by removing the solutions from the top medium reservoirs and adding 150 µl of NMM. For functional studies, PFF treatment on circular tripartite networks was applied to one compartment at DIV 49 or 56 to obtain a final concentration of 10 µg/ml. This was conducted by removing all the media from the top medium reservoirs^29,31^ and only 25 µl of the media from the bottom cell chambers (one third of the cell chamber media) and subsequently applying 50 µl of PFF with a concentration of 15 µg/ml to not disturb the cells (final concentration was 10 µg/ml). Control cultures were treated similarly but with PBS vehicle solution. After 24 h of treatment, the PFF and PBS solutions were washed by removing the solutions from the media reservoirs and adding 100 µl of BrainPhys medium. Thereafter, the media for all the cultures were changed regularly three times per week. The timeline of the PFF treatment and subsequent functional assessments are presented in Supplementary Fig. 1.

### Image acquisition for quantification of α–s aggregation, presynaptic protein content and neurite complexity

Image acquisition for quantification was conducted in a single experiment and was performed on the cultures fixed at 3, 6 and 13 dpt. Acquisition was performed semisupervised by initially choosing a starting region with optimal axonal identification and assemblies labeled by pS129 and then applying automated tile scanning surrounding the starting region (tile size was 0.118 × 0.118 mm) by LSM780 laser scanning confocal microscopy. This approach minimized the user bias during image acquisition while including several tiles without any labeled α–s assemblies in the analysis. All quantified data including α-s aggregation, presynaptic content and neuritic branching, was normalized to neuritic area. This was calculated from constructed 3D neuritic volumes using the Imaris Surface function (Supplementary video 4; Supplementary Fig. 5A and B). This normalization ensured that values were more comparable regardless of the alterations in neuritic morphology during the experiment. Constructed volumes for neurites smaller than 1 µm³ were excluded to mitigate the influence of imaging artifacts.

The number of tiles obtained for different sample groups were prepared for quantification of α-s aggregation as follows: 3 dpt control, 6 regions *n* = 34 tiles; 3 dpt PFF treated, 6 regions *n* = 36 tiles; 6 dpt control, 5 regions *n* = 31 tiles; 6 dpt PFF treated, 6 regions *n* = 24 tiles; 13 dpt control, 5 regions *n* = 29 tiles; 13 dpt PFF treated, 6 regions *n* = 29 tiles, and 3 additional regions, *n* = 19, were also acquired for presynaptic protein quantification for 6 dpt PFF. Details for tile information for each sample group are given in Supplementary Table 3.

Prior to quantification, deconvolution was performed on confocal tile images with Huygens Essential v23.04 (Scientific Volume Imaging, The Netherlands, http://svi.nl) with standard settings. 3D reconstruction and quantification were achieved with the Imaris v9.5 (Oxford Instruments, https://imaris.oxinst.com/) surface detection tool on the images. α–s assemblies were detected and automatically reconstructed with the *Surface* tool. In summary, the foreground intensity levels were initially set to values greater than 5 (ranging from 0 to 255). Surfaces were reconstructed, and volumes outside the range of 1 µm³ to 100 µm³ were excluded from further analysis. The *Intensity Mean*, representing the average intensity within each voxel of a detected surface, was set to 22, and the *Intensity Center*, indicating the intensity of the voxel at the center of a detected surface, was set to 100. These parameter settings were chosen to emphasize serpentine-like aggregates with high fluorescence intensities. The number of aggregates and their total size per tile were calculated. The tiles that did not present any aggregation were shown as zero in the analysis.

The quantification of presynaptic proteins was conducted by utilizing Imaris *Spots* function. The sizes of the *spots* were set to 0.5 µm in diameter on the x and y axes and 1.0 µm on the z axis. The images that did not have sufficient quality to construct the presynaptic *Spots* were discarded from the analysis. The final number of tiles analyzed for different sample groups was as follows: 3 dpt control, *n* = 27 tiles; 3 dpt PFF-treated, *n* = 30 tiles; 6 dpt control, *n* = 31 tiles; 6 dpt PFF-treated *n* = 33 tiles; 13dpt control, *n* = 27 tiles; and 13dpt PFF-treated, *n* = 23 tiles.

Neurite complexity was calculated using the *Filament Tracer* function of Imaris, as described in the previous study^54^, using the same tiles utilized for presynaptic quantification. Branching of neurites were assessed by normalizing the total count of neuritic branching to the total neuritic area coverage. Average pixel intensity of βIII-tubulin was also calculated inside the calculated neuritic area of the constructed neurites.

### Cytotoxicity analyses

The LIVE/DEAD viability kit for mammalian cells (Thermo Fisher Scientific) was used to study the cytotoxic effect of PFF during the experiments. The experiment was conducted in two independent repeats, with a total of 16 image fields analyzed for each group for each time point, except for the 13 dpt PBS-treated group, where a total of 15 image fields were analyzed. The viability test was performed for cultures 3, 6 and 13 dpt following the PFF exposure. The live cells were visualized with 0.1 mM Calcein-AM (emission at 488 nm) and dead cells with 0.5 mM ethidium homodimer-1 (EthD-1, emission at 568 nm, both from Thermo Fisher Scientific). After the treatment, cultures were incubated for 30 min at +37 °C and imaged immediately with Leica Dmi8 Widefield Fluorescence Microscope (Leica, Germany). Cell aggregation is commonly seen in hiPSCs-derived neuronal cultures with the aging^55^, making quantification of live cells challenging. EthD-1 positive death cells were still separately detectable and more reliable for cell viability quantification. Thus, areas of the dead cells as well as number of the dead cells were quantified with CellProfiler software (v.3.1.8)^56^.

### Mitochondrial analysis

Mitochondrial imaging was performed by tracking red fluorescent dye that stained active mitochondria in neurons (MitoTracker Red CMXRos, M7512 Invitrogen). Cultures were loaded with 25 nM MitoTracker for 45 min following an immediate PBS wash. Sequential images were acquired every 90 s for 30 min by using an EVOS FL Auto imaging system (Thermo Fisher Scientific). The experiment was conducted in two independent repeats except for 3 dpt time point for control group. The total number of recordings for each group was as follows: *n* = 3 for 3 dpt control; *n* = 13 for 3 dpt PFF treated; *n* = 6 for 6 dpt control; *n* = 12 for 6 dpt PFF treated; *n* = 8 for 13 dpt control; and *n* = 9 for 13 dpt PFF treated.

For motility analysis, Imaris (Oxford Instruments) software was used to detect motile mitochondria as *spots* and axonal paths as *tracks*. Background subtraction was performed. The radius of the *spots* to be detected was set above 0.2 µm to avoid misdetection due to artifacts. Track straightness was also considered to ensure the detection of straight movements of mitochondria and was calculated as the ratio of track displacement and track length. Track straightness was set above 0.33. Motility was represented by displacement delta length, which indicated the length of the position difference of each detected mitochondrion to its previous position every 90 s. Also, total area covered by the MitoTracker signal was quantified by setting a threshold between 10–20 (ranging from 0 to 255) that discriminates the mitochondrial fluorescence from the background (Fig. 5d). Mitochondrial density was estimated by dividing the number of mitochondria to calculated area (Fig. 5f).

### Calcium imaging

Calcium imaging was performed on hiPSC-derived cortical neurons 3, 6 and 13 dpt. Before imaging, the cells were washed with 4 µM Fluo-4-AM (Abcam, ab241082) in extracellular solution (ECS, 137 mM NaCl, 20 nM HEPES, 5 nM KCl, 5 mM D-glucose, 2 mM NaHCO3, 2 mM CaCl2, 1.2 mM MgCl2, 1 mM Na-pyruvate, and 0.44 mM KH2PO4) for 30 min at +37 °C. After Fluo-4-AM loading, the cells were washed twice with ECS, first with a quick wash followed by a 30 min wash at +37 °C. One-minute time-lapse recordings with 0.28 s intervals were acquired with a Leica Dmi8 Widefield Fluorescence Microscope using 15 ms excitation and 10% laser power. The experiment was conducted in two independent repeats. The total number of recordings for each group was as follows: *n* = 10 for 3 dpt control; *n* = 14 for 3 dpt PFF treated; *n* = 16 for 6dpt control; *n* = 16 for 6dpt PFF treated; *n* = 12 for 13dpt control; and *n* = 15 for 13 dpt PFF treated, and calcium oscillations were further quantified using ImageJ v1.53t (https://imagej.nih.gov/ij/)^57^ and MATLAB R2019a v.9.6.0 (The MathWorks Inc., Massachusetts, United States) software.

Image stacks were analyzed using ImageJ. An additional plugin, Time Series Analyzer V3 (TSA) (https://imagej.nih.gov/ij/plugins/time-series.html), was also used to facilitate data gathering. In ImageJ, all the neuronal soma in each imaged region were semiautomatically detected from one frame of the image stack: the image gray level was estimated by setting the threshold to less than 50 (ranging from 0 to 255) for the default *threshold* function. Regions of interest (ROIs) representing every neuronal soma were automatically placed at each of the locations using the *Analyze Particles* function of ImageJ. Here, the size of active locations was further filtered to a minimum value of 20–30 square pixels to prevent misdetection of smaller particles other than neurons. Another ROI was added manually from a region without any fluorescence signal from biological components to measure the background intensity in the images. After this, the *Get Average* function of TSA was used to export data from each location of the whole image stack.

The obtained data were processed for statistical analysis using a custom MATLAB code. First, the background signal was subtracted from the raw data, and after this, the signal was filtered with a 10-point moving average filter to remove fast frequency noise. Then, ΔF/F0 was calculated, where the signal intensity change ΔF was divided by the base fluorescence level F0, and F0 was approximated to be the mean of the lowest 20% of the signal. Next, a normalization with a linear regression of the signal was performed to account for overall down- or upward trends in the signal intensity across the imaged duration^58^. Peaks from the signal were found with the *findpeaks* MATLAB function using a peak prominence threshold of 0.05 units. The average peak heights and prominences as well as the duration of peaks, distance of peaks from each other, and peak count were stored for analysis. Neurons that had less than 3 detected peaks during the recording were discarded. MATLAB code for the analysis is available at https://github.com/VVuolanto/Calcium-Imaging.

### Microelectrode array measurements and data analysis

Extracellular recordings were obtained in two independent experiments, with each experiment conducted using two different MEA systems: for conventional MEAs Axion Maestro (Axion Biosystems, Atlanta, GA, USA) was used and for circular tripartite MEAs MEA 2100 (MCS, Multichannel Systems GmBH) was used. The Axion Maestro system is controlled by AxIS software with a 12.5 kHz sampling rate. Cells were plated either conventionally on CytoView MEA 48 (Axion Biosystems) or custom-made circular tripartite MEAs^31^. CytoView MEA 48 plates contained 16 electrodes (⌀ 50 μm) per well, and custom circular tripartite MEAs contained 24 (⌀ 30 μm) electrodes per compartment. Recordings were performed under 37 °C temperature control, and a 5% CO_2_ atmosphere was provided during measurements exceeding 10 min. Spontaneous activity was measured twice a week for 10 min for a total of 49–56 days before the PFF treatment and after that up to 13 dpt.

For pharmacological stimulations, 10 min of baseline activity was measured, followed by a 10 min treatment follow-up. Pharmacological experiments were performed at 3, 6 and 13 dpt. The pharmacological reagents were prepared in BrainPhys media at higher concentrations and added in 30 or 100 µl volumes to the proximal compartment of circular tripartite devices after removing 30 µl from the media reservoir or emptying the entire media reservoir, respectively, and 100 µl volumes were added to Axion MEA wells directly to obtain the desired final concentrations. Instantaneous washing between different pharmacological stimulation steps includes emptying the entire media reservoir in circular tripartite devices.

Electrodes that present tonic spiking, artifacts or noise are removed from analysis for the recordings. Spike detection was performed according to the stationary wavelet transform-based Teager energy operator (SWTTEO) algorithm^59^. The mean firing rate was calculated by the R package after discarding the electrodes showing less than 10 spikes per minute for Axion MEAs and 1 spike per minute for custom-made circular tripartite MEAs. The algorithm was implemented in a custom-made MATLAB (MathWorks) script^60^ (https://doi.gin.g-node.org/10.12751/g-node.wvr3jf/).

### Pharmacological stimulations and multiparametric approach

A series of pharmacological stimulations were employed to modulate glutamatergic receptors in the conventional networks or proximal compartments of the circular tripartite networks in two independent experiments. For each pharmacological stimulation (input), recordings that were considered separate responses (output) of the neuronal population were obtained. To assess the data, a multiparametric approach was used, in which these responses were treated as distinct features in the feature vector based on their input/output relationships. The values of features were calculated as a percent change from the previously calculated activity to observe the conditional effect. The procedure was as follows:

i. Baseline recording 1 (10 min)
ii. Application of 50 µM N-methyl-D-aspartate (NMDA, M3262, Sigma) + 100 µM glycine (G8898, Sigma) and recording (10 min)
  *Feature 1:* % change in mean neuronal firing with NMDA + glycine compared to baseline 1.
iii. An instantaneous wash was performed by emptying the entire media reservoir before the next step.
iv. Application of 50 µM D-(-)-2-amino-5-phosphonopentanoic acid (D-AP5, 0106, Tocris) and recording (10 min)
  *Feature 2:* % change in mean neuronal firing with D-AP5 compared to NMDA and glycine.
v. Washing and incubating for 60–90 min
vi. Baseline recording 2 (10 min)
vii. Application of 50 µM S-α-amino-3-hydroxy-5-methyl-4-isoxazolepropionic acid (AMPA, 0254, Tocris) and recording (10 min)
  *Feature 3:* % change in mean neuronal firing with AMPA compared to baseline 2.
viii. An instantaneous wash was performed by emptying the entire media reservoir before the next step.
ix. Application of 50 µM 6-cyano-7-nitroquinoxaline-2,3-dione disodium (CNQX, 1045, Tocris) and recording (10 min)

*Feature 4:* % change in mean neuronal firing with CNQX compared to AMPA.

Separate assessment of neuronal populations in proximal and distal compartments of the circular tripartite network provided 8 additional features by tripling the feature space dimension to 12 (4 features for proximal and 8 features for two separate distal compartments). For visualization, the first three components of principal component analysis (PCA) are presented. The data were prepared by applying mean padding for missing values and standardizing it, ensuring a mean of 0 and a standard deviation of 1 using GraphPad Prism v.9.0 (GraphPad Software, Boston, Massachusetts USA). This process removed any potential bias or scale discrepancies by also keeping the integrity of the dataset’s statistical properties.

The data were visualized by using an inhouse MATLAB script that included the creation of 3D scatter plots for the control and treatment groups based on three principal component (PC) values. Different colors were assigned to each class of data points. Two surfaces were generated based on the data points of each class using the *meshgrid* function of MATLAB, and color coding was performed based on their respective classes. The degree of overlap between the two groups was quantified in a 2D projection by calculating the percentage of the smaller surface to the intersection.

### qRT-PCR analysis

RNA samples were collected in a single experiment from 2 to 3 wells pooled together for both control and PFF treated neuronal cultures at the 3, 6 and 13 dpt. RNA was isolated with a NucleoSpin RNA kit (Macherey-Nagel, Düren, Germany) according to manufacturer’s protocol. The concentration and purity of RNA were quantified with a NanoDrop 1000 (Thermo Fisher Scientific). RNA was converted to cDNA using a High Capacity cDNA Reverse Transcription Kit (Thermo Fisher Scientific). The expression levels of NMDAR, AMPAR and KAR subunits were analyzed (Supplementary Table 1) with TaqMan assays using an Quantstudio FLexreal-time PCR system (Thermo Fisher Scientific) (Supplementary Table 1). Each 12 µl reaction contained 10 ng cDNA, 0.5 µl 20 × TaqMan Gene Expression Assay and 5 µl 2 × TaqMan Gene Expression Master Mix. The data were analyzed by calculating the $\Delta \Delta {\rm{C}}_{T}$ method using Glyceraldehyde-3-phosphate dehydrogenase (GAPDH) as an endogenous control and all samples were normalized to 3 dpt control sample. All the samples were run in triplicate, and data are presented with their average.

### Statistical analysis

All statistical analysis were performed with SPSS software v.28.01.0 (IBM Corp., Armonk, NewYork, USA) using nonparametric tests, due to the non-Gaussian distribution of the data. Tampere University statistician Heini Huhtala was consulted for the statistical tests. Mann-Whitney U test was used for comparing independent data from control and PFF treated cultures at same time points. Wilcoxon Signed Ranked test were used for analyzing repeated samples including MEA firing rate changes according to baseline. Kruskal-Wallis test was used to compare mean ranks of aggregation quantities in PFF group and control group at 3, 6 and 13 dpt as well as from 3 to13 dpt in the PFF group. Dunn’s post-hoc test was performed with Bonferroni correction for multiple comparisons after Kruskal-Wallis test.

### Supplementary information

Supplementary Materials

Supplementary movie 1. 3D visualization of neuritic bundles and PFF-induced α-s aggregation.

Supplementary movie 2. Video showing mitochondrial displacement.

Supplementary movie 3. Video showing calcium activity.

Supplementary movie 4. 3D-reconstruction of neuronal networks.

## Data Availability

The datasets generated and/or analyzed during the current study are available from the corresponding author upon request. Aligning with Creative Commons Attribution (CC BY) licensing, we require that users of data provide appropriate credit to authors, refer to the original work and indicate any changes made.

## Code availability

The manuscript provided references and/or repository addresses for the codes used in the study. Matlab codes for the calcium and MEA analysis is located at https://github.com/VVuolanto/Calcium-Imaging and https://gin.g-node.org/NeuroGroup_TUNI/Comparative_MEA_dataset/src/master/Codes, respectively. The plugin to analyze image stacks, Time Series Analyzer V3 (TSA) is located at https://imagej.net/ij/plugins/time-series.html.
