# Supplementary material for: Human tripartite cortical network model for temporal assessment of alpha-synuclein aggregation and propagation in Parkinson’s Disease
Source: NPJ Parkinsons Dis. 2024 Jul 28;10:138. doi: 10.1038/s41531-024-00750-x (PMC11284226; doi:10.1038/s41531-024-00750-x)
Supplement: Supplementary file 1 — Supplementary Materials [file 41531_2024_750_MOESM1_ESM.pdf]

Supplementary Tables

Supplementary Table 1: Analyzed genes with used primers in qRT-PCR*

iGluR Subtype	Receptor subunit	Genes symbol	Taqman assay ID
NMDAR	GluN1	GRIN1	Hs00609561_m1
	GluN2A	GRIN2A	Hs00168219_m1
	GluN2B	GRIN2B	Hs01002012_m1
	GluN2C	GRIN2C	Hs01016628_m1
	GluN2D	GRIN2D	Hs00181352_m1
	GluN3A	GRIN3A	Hs00370290_m1
	GluN3B	GRIN3B	Hs00879911_g1
AMPAR	GluA1	GRIA1	Hs00181348_m1
	GluA2	GRIA2	Hs01564838_m1
	GluA3	GRIA3	Hs01557464_m1
	GluA4	GRIA4	Hs00898779_m1
KAR	GluK1	GRIK1	Hs00543710_m1
	GluK2	GRIK2	Hs00222637_m1
	GluK3	GRIK3	Hs00168182_m1
	GluK4	GRIK4	Hs00205979_m1
	GluK5	GRIK5	Hs00361585_m1

* GAPDH was used as housekeeping gene (Taqman assay ID: Hs99999905_m1)

Supplementary Table 2: The details of tripartite chips used for each experiment.

				SPONTANEOUS RESPONSE (DAILY BASELINES)										RESPONSES TO GLUTAMATERGIC RECEPTOR MODULATORS											
				PROXIMAL (1 compartment)					DISTAL (2 compartments)					PROXIMAL (1 compartment)						DISTAL (2 compartments)					
	MEA #	ND	BL	3-dpt	6-dpt	7/8-dpt ✪	10-dpt	13-dpt	3-dpt	6-dpt	7/8-dpt	10-dpt	13-dpt	3-dpt NMDA	3-dpt AMPA	6-dpt NMDA	6-dpt AMPA	13-dpt NMDA	13-dpt AMPA	3-dpt NMDA	3-dpt AMPA	6-dpt NMDA	6-dpt AMPA	13-dpt NMDA	13-dpt AMPA
EXPERIMENT 1	*1*	✓	✓	✓	✓	✓	✓	✓	✓✓	✓✓	✓✓	✓✓	✓✓					✓	✓					✓✓	✓✓
	2	✓	✓	✓	✓	✓	✓	**	✓✓	✓✓	*✓	*✓	**					**	**					**	**
	3	✓	✓	✓	✓	✓	✓	✓	✓✓	✓✓	✓✓	✓✓	✓✓					✓	✓					✓✓	✓✓
	4	✓	✓	✓					✓✓					✓	✓					✓✓	✓✓				
	5	✓	✓	✓	✓				✓✓	✓✓						✓	✓					✓✓	*✓		
	6	✓	✓	✓	✓	✓	✓	✓	*✓	✓✓	✓✓	✓✓	✓✓					✓	✓					✓✓	✓✓
	7	✓	✓	✓					✓✓					✓	✓					✓✓	✓✓				
	8	✓	✓	✓					*✓					✓	✓					*✓	✓✓				
	9	✓	✓	✓					✓✓					✓	✓					✓✓	✓✓				
	10	✓	✓	*					*					*	✓					*	*✓				
	11	✓	✓	✓	*				✓✓	*						*	✓					*	✓✓		
	12	✓	✓	✓	✓	✓	✓		✓✓	✓✓	✓✓	✓✓	**												
	13	✓	✓	✓	✓				✓✓	✓✓						✓	✓					✓✓	✓✓		
	14	✓	✓	✓	✓				✓✓	✓✓						✓	✓					✓✓	✓✓		
	15	✓	✓	✓	✓				*✓	✓✓						✓	✓					✓✓	✓✓		
EXPERIMENT 2	*16*	✓	✓	✓	✓				✓✓	✓✓						✓	✓					✓✓	✓✓		
	17	✓	✓	✓		✓	✓	✓	✓✓		✓✓	✓✓	✓✓					✓	✓					✓✓	✓✓
	18	✓	✓	✓		✓	✓	✓	✓✓		✓✓	✓✓	✓✓					✓	✓					✓✓	✓✓
	19	✓	✓	✓					✓✓					✓	✓					✓✓	*				
	20	✓	✓	✓		✓	✓	✓	✓✓		✓✓	✓✓	✓✓					✓	✓					✓✓	✓✓
	21	✓	✓	✓		✓	✓	✓	✓✓		✓✓	✓✓	✓✓					✓	✓					✓✓	✓✓
	22	✓	✓	✓					✓✓					✓	✓					✓✓	✓✓				
	23	✓	✓	✓					✓✓					✓	✓					✓✓	✓✓				
	24	✓	✓	✓		✓	✓	✓	✓✓		✓✓	✓✓	✓✓					✓	✓					✓✓	✓✓
	25	✓	✓	*	✓				✓✓	✓✓						✓	✓					✓✓	✓✓		
	26	✓	✓	✓	✓				✓✓	✓✓						✓	✓					✓✓	✓✓		
	27	✓	✓	✓					✓✓					✓	✓					✓✓	✓✓				
	28	✓	✓	✓					✓✓					✓	✓					✓✓	✓✓				
	29	✓	✓	✓		*	✓	✓	*✓		*✓	✓✓	✓✓					*	✓					*	✓✓
	30	✓	✓	✓	✓				✓✓	✓✓						✓	✓					✓✓	✓✓		
	31	✓	✓	✓	✓				✓✓	✓✓						✓	✓					✓✓	✓✓		
	32	✓	✓	✓	✓				✓✓	✓✓						✓	✓					✓✓	*✓		
	**(n)**	**32**	**32**	**30**	**15**	**10**	**11**	**9**	**58**	**30**	**20**	**21**	**18**	**9**	**10**	**10**	**11**	**8**	**9**	**17**	**17**	**20**	**20**	**16**	**18**

Red and blue boxes indicate PFF treated and control circular tripartite models, respectively. Black boxes are MEAs which were not recorded in that time point (used/reserved for another time point).

single tick (✓): proximal compartment recorded and used for analysis, **double thick (✓✓):** both distal compartments recorded and used for analysis **one asterix and single tick (*✓) :** one of the distal compartments recorded and used for analysis**,** and **one asterix (*):** both distal compartments discarded from analysis **(**):** MEA was totally damaged/not recorded. Unused or discarded recordings were because of technical failure during the recording, noise or because of damaged MEA.

✪ MEAs recorded day 7 were not recorded on day 8 and vice versa. Day 7 and 8 recordings were pooled/analyzed together.

ND: Network development analysis

BL: Baseline recordings before PFF treatment

Supplementary Table 3: Details for confocal microscopy tiles used for quantifying presynaptic protein content and α–s aggregation.

Time point	Treatment	Number of Regions	Number of Tiles (n)*	Discarded tiles for presynaptic content analysis ***
3-dpt	PBS	6	34	7
	PFF	6	36	6
6-dpt	PBS	5	31	0
	PFF	4 + 3 **	24 + 19**	10
13-dpt	PBS	5	29	2
	PFF	6	29	6

* Each tile has 0.118 x 0.118 mm size taken in the vicinity of starting regions
** 3 additional regions N=19 tiles taken for presynaptic protein content quantification
*** Tiles are discarded due to the low image quality or image artefacts to construct accurate presynaptic *Spots*

Supplementary Figures

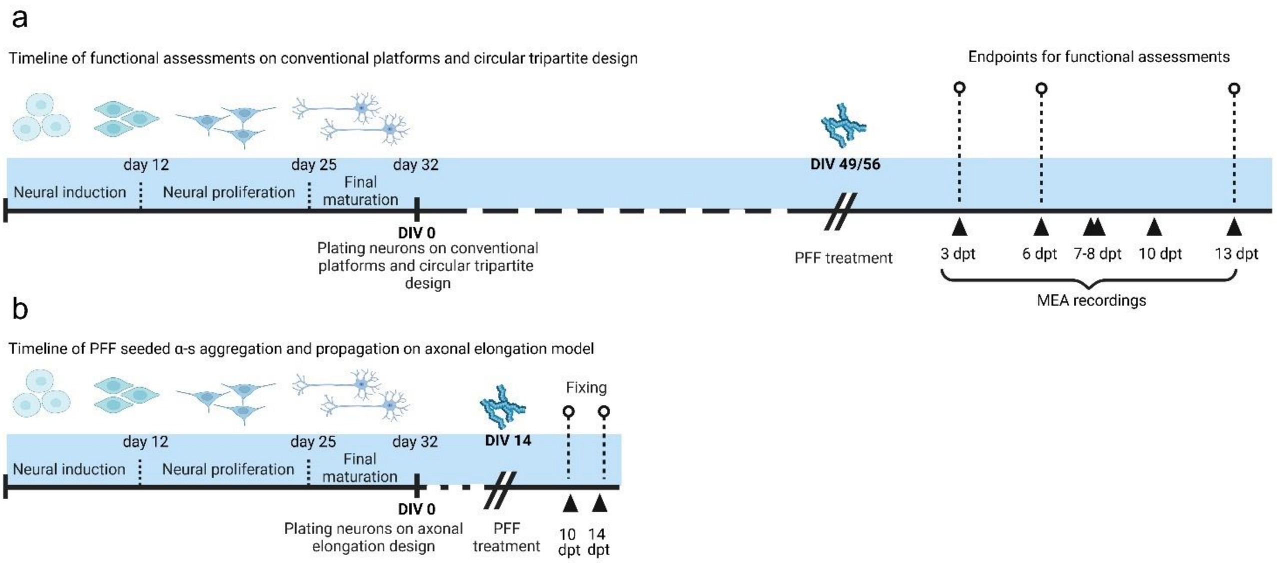

Supplementary Figure 1. Timeline of development of cortical neurons and PFF seeding experiments. Differentiation and maturation of cortical neurons before plating on final culturing platforms includes neural induction stage (first 12 days), neural proliferation stage (day 13 to day 25) and final maturation stage (day 26 to 32). Cells were cultured on conventional, axonal elongation, or circular tripartite platforms (DIV 0). **a** For functional studies PFF treatment on circular tripartite design was performed at DIV 49 or 56. MEA recordings were obtained 3, 6, 7-8, 10 and 13 days after PFF treatment and glutamatergic modulators were applied on endpoints at 3, 6 and 13 dpt. **d** To test and optimize PFF-induced α-s aggregation and axonal transport, the axonal elongation design was treated with PFF at DIV 14 and aggregation was analyzed at 10 and 14 dpt. Figure was created using Biorender.com.

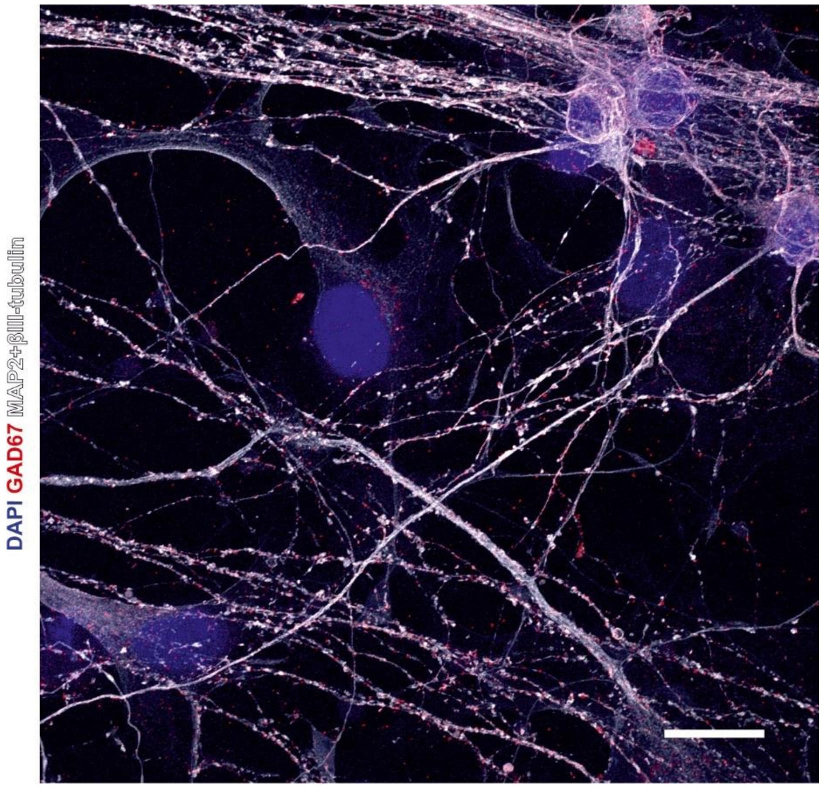

Supplementary Figure 2. GABAergic markers in neuronal cells. Representative image depicting the expression of the GABAergic marker GAD67 in hiPSC-derived cortical neurons at DIV 35, prominently localized within neurites identifiable by MAP2 and βIII-tubulin and is visualized as red puncta. Scale bar is 10μm.

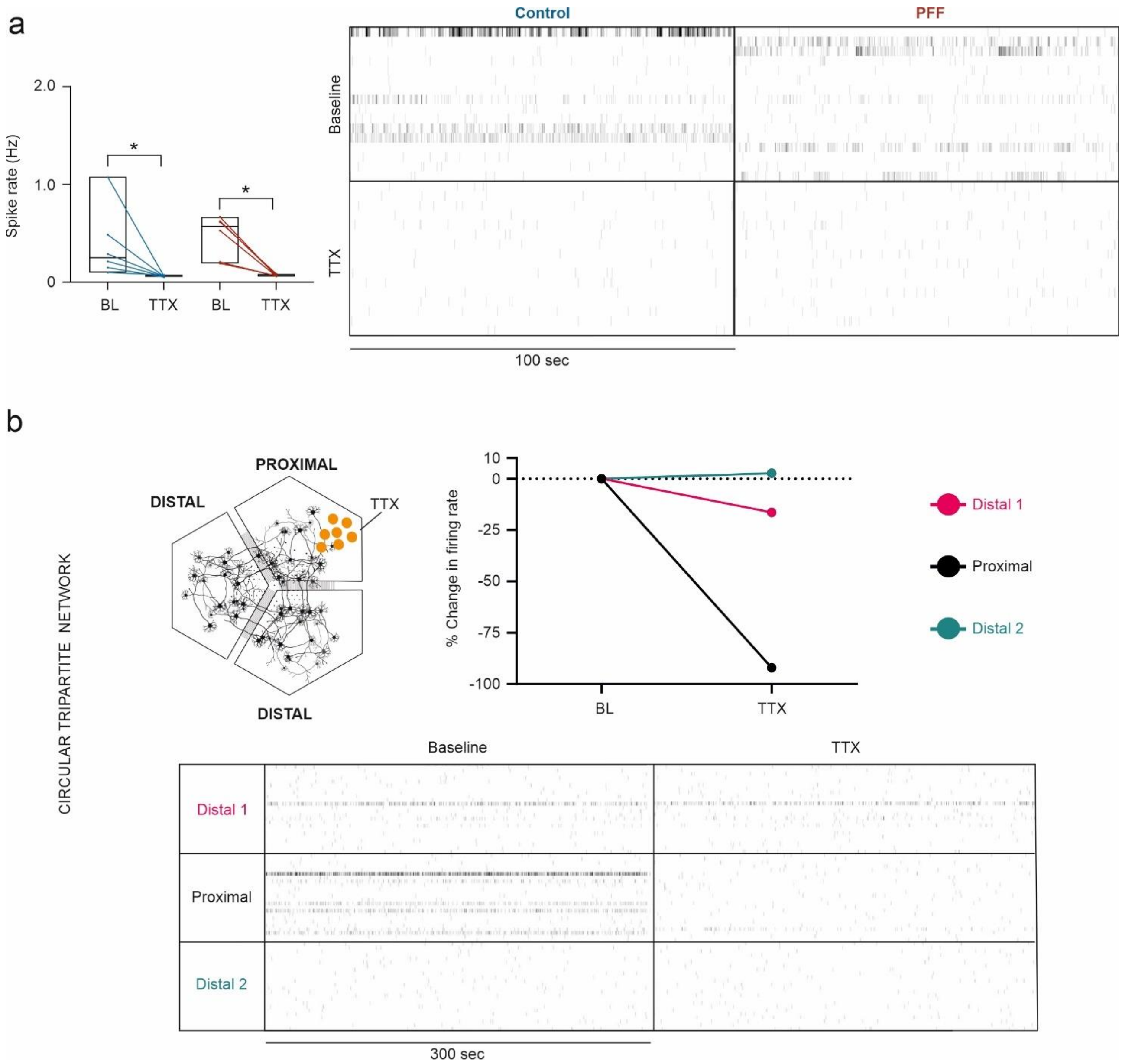

Supplementary Figure 3. Effect of Na$^+$ channel blocker tetrodotoxin on conventional and circular tripartite networks. a Na$^+$ channel blocker tetrodotoxin (TTX) significantly silences the neuronal activity in MEA wells (n=6) in conventional networks. p values were calculated with Wilcoxon Signed Ranked test for pairwise comparison of TTX application to baseline (BL) (*p<0.05). Raster plots represent the neuronal activity before and after TTX application **b** TTX application silenced the neuronal activity majorly in the applied proximal compartment while affecting the distal compartments minimally or not at all. Raster plots represent the proximal and distal compartments of the experimented circular tripartite network. The illustrations were created using Biorender.com.

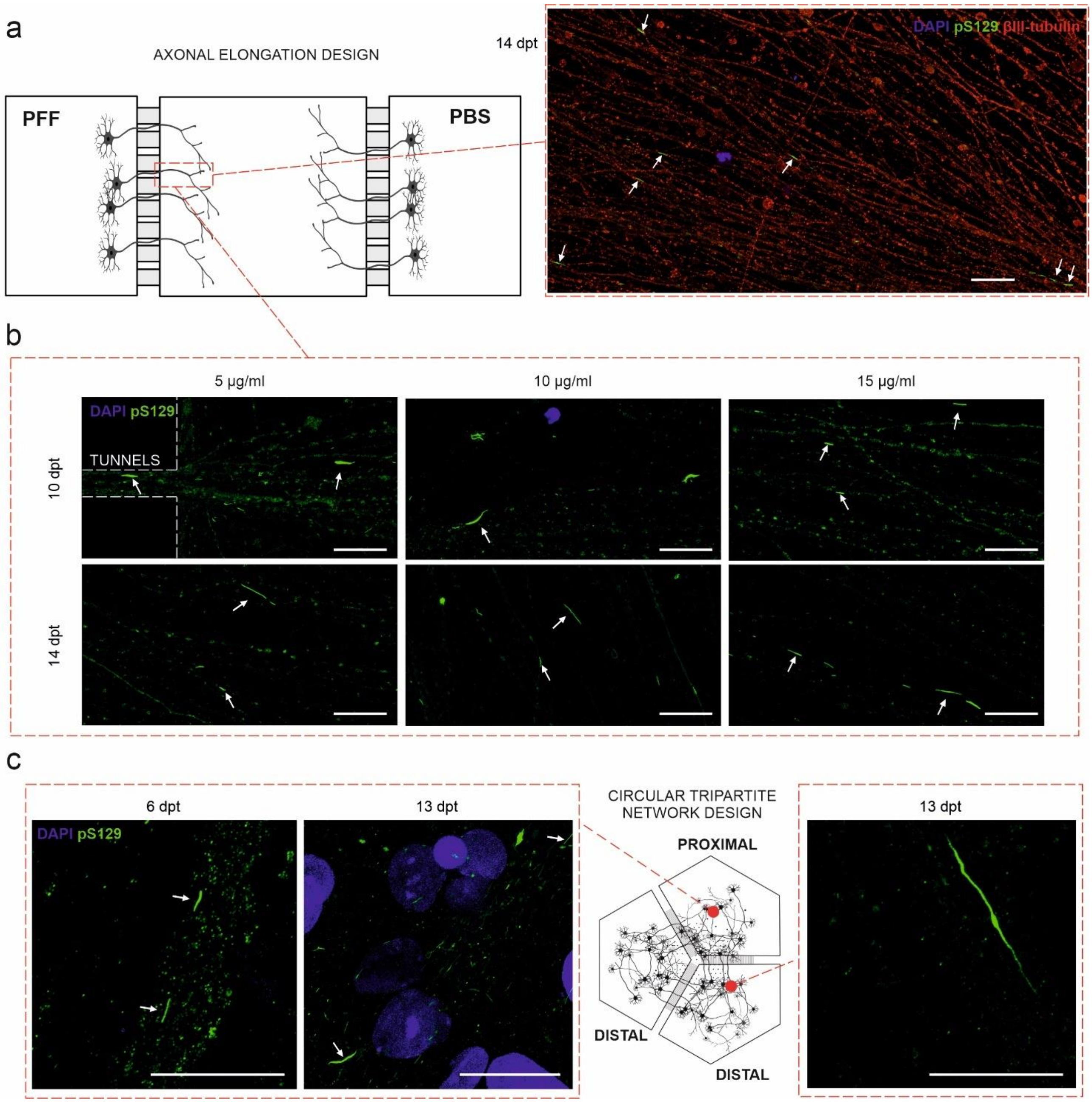

Supplementary Figure 4. PFF seeded α-s aggregation and propagation of aggregated forms with different PFF concentrations. a Representative image from the axon alignment model demonstrate pS129 (green indicated with white arrows) positive α-s aggregates shown in middle compartment close to the PFF treated compartment. Aggregates are aligned with axons (βIII-tubulin, red). Also, **b** pS129 positive α-s aggregates (green indicated with white arrows) observed as a result of PFF seeding exposed in concentrations of 5, 10 and 15 µg/ml at 10 and 14 dpt in middle compartment close to the PFF treated compartment. **c** Representative images from the circular tripartite model showing pS129 positive aggregates (green indicated with white arrows) at 6 and 13 dpt in proximal compartment and at 13 dpt in distal compartment. All the scale bars are 20µm. The illustrations were created using Biorender.com.

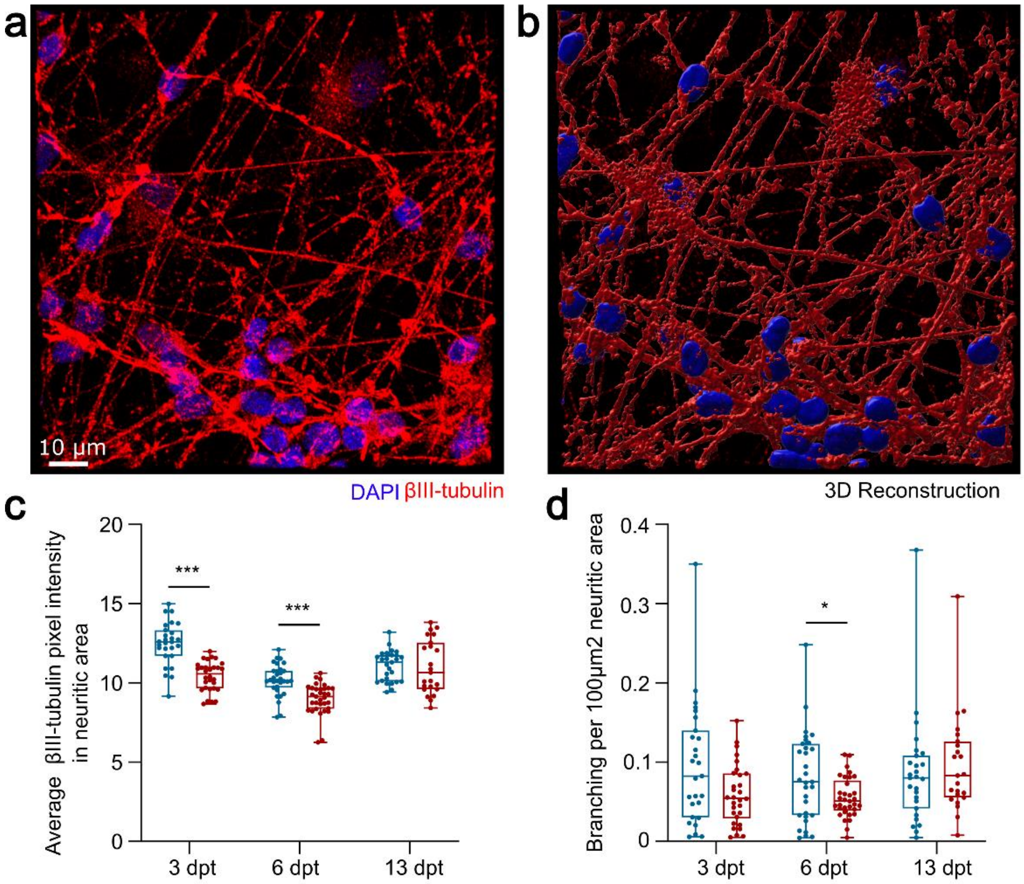

Supplementary Figure 5. Impact of PFF-seeded α-s aggregation on neurite complexity. a βIII-tubulin labeled neuritic areas were used for 3D neurite reconstruction seen in **b** and Supplementary video 4. **c** The average pixel intensity of βIII-tubulin was calculated in the neuritic area. **d** Neurite complexity was assessed by calculating number of branching per 100μm^2 neuritic area. p values were calculated with the Mann-Whitney U test to compare groups at each time point (*p<0.05, **p<0.01 and ***p<0.001).

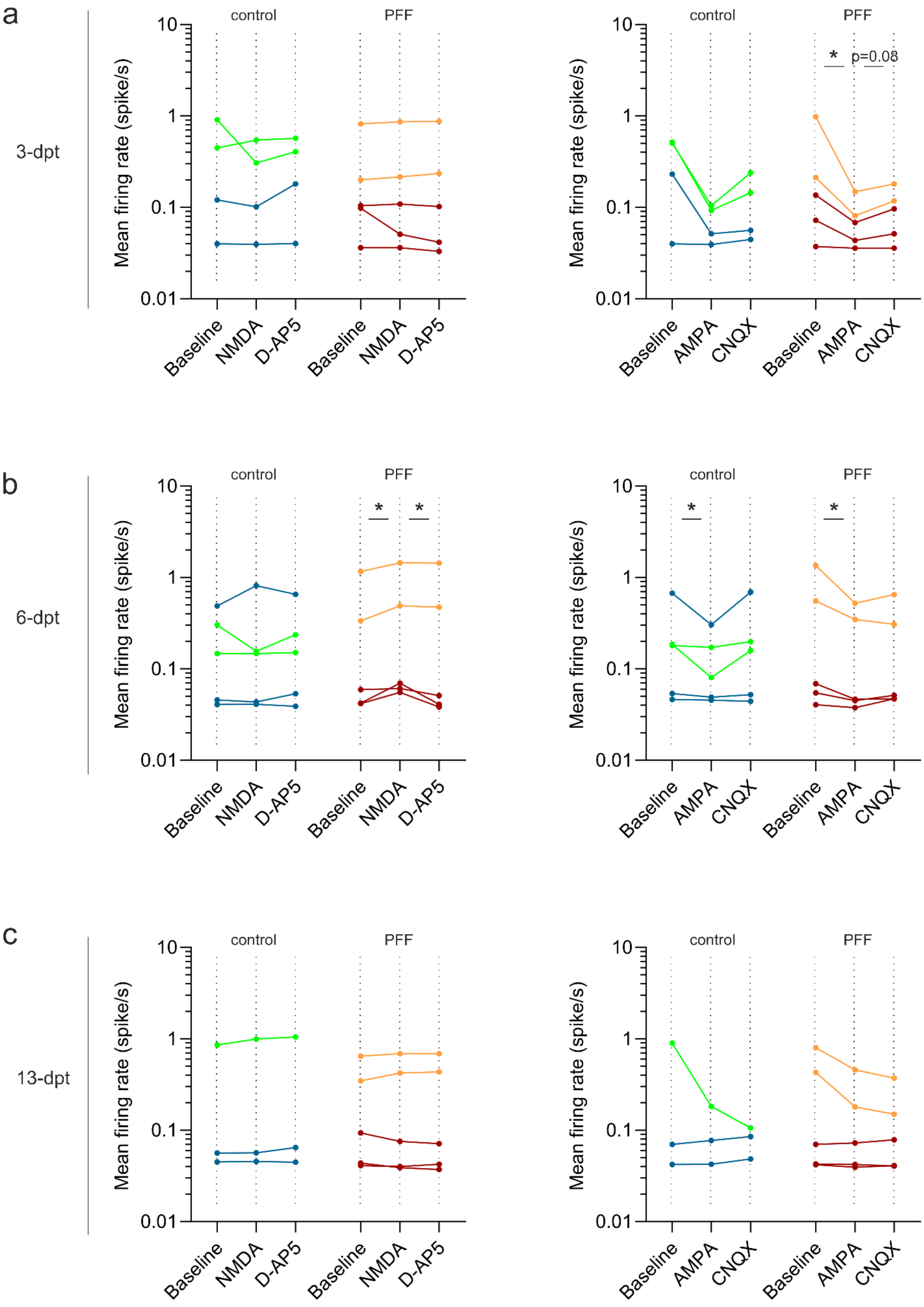
a
3-dpt
b
6-dpt
c
13-dpt
control
PFF
Mean firing rate (spike/s)
10
1
0.1
0.01
Baseline
NMDA
D-AP5
AMPA
CNQX
*
p=0.08

Supplementary Figure 6. Glutamatergic modulator-induced neuronal activity in conventional networks after PFF treatment. Figure demonstrates the data from Fig. 8b-e as absolute firing rates for recordings from proximal compartments at **a** 3 dpt **b** 6 dpt and **c** 13 dpt. Green-orange and blue-red color combinations denote control-treatment pairs in the first and second experiments, respectively. p values were calculated with the Wilcoxon Signed Ranked test for pairwise comparisons (*p<0.05).

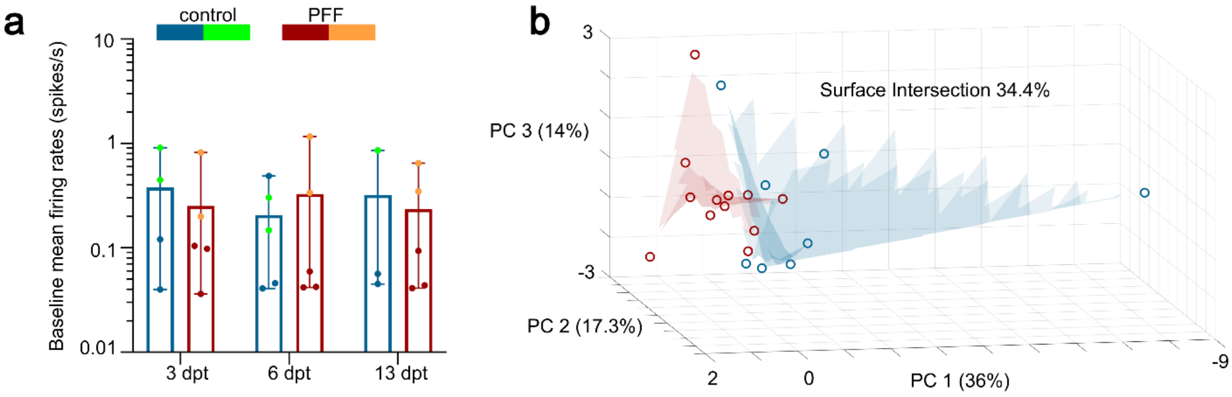

Supplementary Figure 7. a Baseline mean firing rates were recorded from proximal compartments prior to serial glutamatergic modulator stimulation. Green-orange and blue-red color combinations denote control-PFF treatment pairs in the first and second experiments, respectively. No significant change is observed between groups (Mann-Whitney U test). **b** 3D representation of multiparametric analysis presented with the first three principal components (PC1-3) and calculated intersection percentages between the surfaces of control and PFF treated network values. Data were pooled from 3 dpt and 6 dpt time points and each data point in the image represents values obtained from a circular tripartite network.

Supplementary videos

Supplementary movie 1. 3D visualization of neuritic bundles and PFF-induced α-s aggregation. The recruited/aggregated forms of α-s (green) are phosphorylated at residue S129 (pS129). These serpentine-like aggregates are colocalized with neuritic bundles labeled with βIII-tubulin (purple).

Supplementary movie 2. Video showing mitochondrial displacement. Mitochondrial spots and total area covered by the MitoTracker signal are presented in time-lapse images. The video, with a total length of 30 minutes, is composed of 20 frames with 90 seconds between each frame.

Supplementary movie 3. Video showing calcium activity. The changes in calcium concentration are tracked over time using the fluorescence intensity of the calcium-binding dye Fluo-4 in the

automatically detected neurons (marked with yellow areas). The calcium oscillations recorded from the region within the red circle are presented in Figure 5a.

Supplementary movie 4. 3D-reconstruction of neuronal networks. Neuronal networks were reconstructed using Imaris v9.5 (Oxford Instruments, https://imaris.oxinst.com/) with images obtained via confocal microscopy. Neuritic surface areas were estimated from the reconstructed βIII-tubulin labeled neurites (red).
